# Targeting the anti-apoptotic Bcl-2 family proteins: machine learning virtual screening and biological evaluation of new small molecules

**DOI:** 10.7150/thno.64233

**Published:** 2022-02-28

**Authors:** Elisabetta Valentini, Simona D'Aguanno, Marta Di Martile, Camilla Montesano, Virginia Ferraresi, Alexandros Patsilinakos, Manuela Sabatino, Lorenzo Antonini, Martina Chiacchiarini, Sergio Valente, Antonello Mai, Gianni Colotti, Rino Ragno, Daniela Trisciuoglio, Donatella Del Bufalo

**Affiliations:** 1 Preclinical Models and New Therapeutic Agents Unit, IRCCS Regina Elena National Cancer Institute, Via Elio Chianesi 53, Rome, Italy.; 2 Department of Chemistry, Sapienza University of Rome, Piazzale Aldo Moro 5, Rome, Italy.; 3 Sarcomas and Rare Tumours Departmental Unit- IRCCS Regina Elena National Cancer Institute, Via Elio Chianesi 53, Rome, Italy.; 4 Rome Center for Molecular Design, Department of Drug Chemistry and Technology, Sapienza University of Rome, Piazzale Aldo Moro 5, Rome, Italy.; 5 Department of Drug Chemistry, Sapienza University of Rome, Piazzale Aldo Moro 5, Rome, Italy.; 6 Pasteur Institute, Cenci Bolognetti Foundation, Sapienza University of Rome, Piazzale Aldo Moro 5, Rome, Italy.; 7 Institute of Molecular Biology and Pathology, Italian National Research Council, Piazzale A.Moro 5, 20 Rome, Italy.

**Keywords:** Bcl-2 family, Bcl-2 family inhibitors, virtual screening, apoptosis, autophagy

## Abstract

Bcl-2 family anti-apoptotic proteins are overexpressed in several hematological and solid tumors, and contribute to tumor formation, progression, and resistance to therapy. They represent a promising therapeutic avenue to explore for cancer treatment. Venetoclax, a Bcl-2 inhibitor is currently used for hematological malignancies or is undergoing clinical trials for either hematological or solid tumors. Despite these progresses, ongoing efforts are focusing on the identification and development of new molecules targeting Bcl-2 protein and/or other family members.

**Methods:** Machine learning guided virtual screening followed by surface plasmon resonance, molecular docking and pharmacokinetic analyses were performed to identify new inhibitors of anti-apoptotic members of Bcl-2 family and their pharmacokinetic profile. The sensitivity of cancer cells from different origin to the identified compounds was evaluated both in *in vitro* (cell survival, apoptosis, autophagy) and *in vivo* (tumor growth in nude mice) preclinical models.

**Results:** IS20 and IS21 were identified as potential new lead compounds able to bind Bcl-2, Bcl-xL and Mcl-1 recombinant proteins. Molecular docking investigation indicated IS20 and IS21 could bind into the Beclin-1 BH3 binding site of *wild type* Bcl-2, Bcl-xL and Mcl-1 proteins. In particular, although the IS21 docked conformation did not show a unique binding mode, it clearly showed its ability in flexibly adapting to either BH3 binding sites. Moreover, both IS20 and IS21 reduced cell viability, clonogenic ability and tumor sphere formation, and induced apoptosis in leukemic, melanoma and lung cancer cells. Autophagosome formation and maturation assays demonstrated induction of autophagic flux after treatment with IS20 or IS21. Experiments with z-VAD-fmk, a pan-caspase inhibitor, and chloroquine, a late-stage autophagy inhibitor, demonstrated the ability of the two compounds to promote apoptosis by autophagy. IS21 also reduced *in vivo* tumor growth of both human leukemia and melanoma models.

**Conclusion:** Virtual screening coupled with *in vitro* and* in vivo* experimental data led to the identification of two new promising inhibitors of anti-apoptotic proteins with good efficacy in the binding to recombinant Bcl-2, Bcl-xL and Mcl-1 proteins, and against different tumor histotypes.

## Background

Over the last decades, a remarkable progress in cancer research and therapy has been achieved, with a better understanding of the apoptotic machinery and the association between tumors and altered expression of proteins involved in apoptosis regulation 1. The members of the B-cell lymphoma-2 (Bcl-2) family are central regulators of apoptosis triggered by a plethora of stress signals. The anti-apoptotic members of this family exert their pro-survival function by binding and inhibiting the pro-apoptotic proteins, the sensors of cellular stress (the BH3-only proteins), and the effectors of apoptosis 1. Protein-protein interaction occurs through the binding between the helical BH3 motifs of pro-apoptotic molecules and a hydrophobic groove on the surface of anti-apoptotic proteins 1. Beyond apoptosis, Bcl-2 family is also implicated in other cellular pathways leading to cell survival or demise as autophagy, a cellular recycling process highly conserved 2.

In many cancers, high levels of anti-apoptotic proteins such as Bcl-2, Bcl-xL and Mcl-1 were shown to contribute, not only to lack of response to chemotherapy, but also to tumor initiation and progression 3. In this regard, we demonstrated that Bcl-2 modulates *in vitro* and *in vivo* tumor progression-associated properties and angiogenesis through regulation of microRNA and transcription factors 4-14. We and other authors also reported evidences regarding the role played by Bcl-xL in cancer progression 11, 15-18.

Targeting anti-apoptotic proteins is a promising therapeutic avenue to explore for cancer treatment. To this purpose, different strategies are under continuous investigation. Among them, we can point to Bcl-2 antisense oligonucleotide, evaluated in phase I and II clinical trials, but with unsatisfactory results 19. Peptides and small molecules to alternatively target the BH4 domain have also been evaluated 20, 21. However, most of the efforts have been made in investigating the therapeutic efficacy of compounds able to displace the BH3 domain binding 3. Nevertheless, two pan inhibitors ABT-737 and ABT-263 (navitoclax), the first *bona fide* BH3 mimetics, resulted in significant toxicities associated with off-target effects 22; among the side effects, the induction of thrombocytopenia 23, 24. These limitations seems to have been overcame with ABT-199 (also known as venetoclax), a potent and selective Bcl-2 binder, approved by the US Food and Drug Administration for the treatment of first-line and relapsed/refractory chronic lymphocytic leukemia (CLL), acute myeloid leukemia (AML) and small lymphocytic lymphoma (SLL) 25, 26. Mechanisms of resistance to ABT-199 in CLL patients have been identified in mutations involving several residues, including 101 and 104 27, 28. Even if Bcl-2 inhibition represents a promising therapeutic approach for solid tumors, the role of ABT-199 remains to be determined in clinical settings 26, 29. Despite these progresses in the field, ongoing efforts are focusing on the identification and development of new molecules targeting Bcl-2 protein and/or other family members.

The aim of this work was to identify new possible inhibitors of anti-apoptotic members of Bcl-2 family. To this purpose, statistical/mathematical models were built through the use of machine learning (ML) techniques capable of predicting and quantifying the biological activity of purchasable small molecule as potential new Bcl-2 inhibitors (virtual screening, VS) 30-34. ChEMBL 35 and PubChem 36 databases were used as source of known biological and structural data to feed the ML methods. Performing this approach, we identified two inhibitors showing high affinity for recombinant Bcl-2, Bcl-xL and Mcl-1 proteins and *in vitro/in vivo* anti-tumoral activity in preclinical cancer models with different histotype.

## Methods

### Computational procedures

All details of computational procedure regarding virtual screening protocol and molecular docking assessment were reported in the Supplementary Information (**Table S1-S18; Figure S1-S7**).

### Cell cultures

Human melanoma M14, Sbcl2 and A375, non-small cell lung cancer (NSCLC) H1299, acute promyelocytic leukemia (APL) HL60 and AML U937 cell lines were cultured in RPMI 1640 medium (Euroclone, Milan, IT) supplemented with 10% fetal bovine serum (Hyclone, Thermoscientific, South Logan, UT), 1% L-glutamine (Euroclone) and 100 μg/ml penicillin/streptomycin (Euroclone). Human colon HCT116 and breast MDA-MB-231 cancer cell lines were cultured in DMEM medium (LONZA, Verviers, Belgium) supplemented with 10% fetal bovine serum, 1% L-glutamine and 100 μg/mL penicillin/streptomycin. M14 and H1299 stably overexpressing EGFP-LC3B or mRFP-EGFP-LC3B were generated as previously reported 37. Cell lines have been recently tested for mycoplasma contamination, and authenticated.

### Reagents preparation

The 57 compounds derived by the VS were purchased by Vitas-M Laboratory (https://vitasmlab.biz/ Radio City, Hong Kong) and by ChemSpace (https://chem-space.com/, Riga, Latvia). All chemicals were of the highest purity available, as certified by vendor, and were dissolved in DMSO (Sigma-Aldrich, St. Louis, Missouri, USA). For all experiments, cells were treated with < 1% DMSO as control (untreated in figures and figure legends). Cancer cells were exposed to compounds, at concentrations ranging from 5 to 50 µM, for 24 or 72 h. Chloroquine diphosphate (CQ, 5 µM for 72 h, Sigma-Aldrich) and z-VAD-fmk (z-VAD, 50 µM for 72 h, Abcam, Cambridge, UK) were dissolved in water and DMSO, respectively.

### Cell viability, clonogenic and tumor sphere formation assays

The effect of compounds on cell viability was performed by measuring 3-[4,5-dimethylthiazol-2-yl]-2,5-diphenyltetrazolium bromide inner salt (MTT, Sigma-Aldrich) dye absorbance (solid tumor cells) or by quantization of ATP present in metabolically active cells using CellTiter-Glo® Luminescent (Promega, Fitchburg, Wisconsin, USA) (hematologic cells) following manifacturer's protocol. To evaluate the cell colony-forming ability, cell suspension from different samples was seeded into 60-mm Petri dishes. After 10 days, colonies were stained with 2% methylene blue in 95% ethanol and counted (1 colony > 50 cells). The surviving fractions were calculated as the ratio of absolute survival of the treated sample/survival of DMSO-treated control sample.

For tumor sphere assay, cells were recovered after treatment, counted and seeded in 24-well ultralow attachment surface plates at a density of 5 × 10^2^ cells/well and cultured as previously described 38. After 10 days, spheres were photographed and counted. The percentage of spheres normalized to the number of seeded cells was calculated.

### Western blot analyses

Western blot analyses of total protein extracts were performed as previously described 6. Immunodetection was performed using antibodies directed to poly (ADP-ribose) polymerase (PARP; BD Biosciences San Diego, CA, USA), LC3B (Cell Signaling Technology, Danvers, MA), HSP72/73 (Calbiochem, EMD Biosciences, La Jolla, CA), Bcl-2 (SC-509, Santa Cruz Biotechnology Inc, Dallas, TX, USA), Bcl-xL (SC-271121, Santa Cruz Biotechnology Inc), Mcl-1 (SC-12756, Santa Cruz Biotechnology Inc) α-tubulin (Santa Cruz Biotechnology Inc) actin (Sigma-Aldrich, St. Louis, Missouri, USA). Anti-rabbit or anti-mouse immunoglobulin G-horseradish peroxidase-conjugated antibodies (Amersham Biosciences, Freiburg, Germany) were used as secondary antibody. Images were acquired by Image Lab Software (Bio-Rad, Hercules, CA, USA), using a ChemiDoc System instrument (Bio-Rad). The densitometric evaluation was performed using Image J software and normalized with relative controls.

### Flow cytometric analysis

Flow cytometric analysis (BD Accuri™ C6, BD Biosciences) was performed to evaluate cell cycle distribution by propidium iodide (PI) staining, and cell viability by using the PI dye exclusion assay as previously reported 38.

### Analysis of Autophagy

Evaluation of autophagosomal structures was obtained by fluorescence microscopy observing LC3B puncta in M14EGFP-LC3B and H1299EGFP-LC3B cells. Autophagosomes and autolysosomes correct fusion was analyzed using H1299 cells expressing mRFP-EGFP-LC3B. Preparation of microscopy slides and analysis of images was performed as previously described 37.

### Surface Plasmon Resonance (SPR) analysis

FastStep experiments were performed with a SensiQ Pioneer apparatus. Bcl-2 immobilization on COOH5 sensorchips was carried out essentially as previously reported 39, 40. All the experiments were carried out at 25 °C in degassed HEPES 20 mM, pH 7.4, NaCl 150 mM, 0.005% surfactant P20 + 2% DMSO (HBSP2%D). Analytes (IS1, IS9, IS20, IS21, IS27, IS29, ISP, ISQ and ABT-199) in HBSP2%D were injected at a flow rate = 100 μL/min, at the following concentrations: 0-30 s: 0.625 μM; 31-60 s: 1.25 μM; 61-90 s: 2.5 μM; 91-120 s: 5 μM; 121-150 s: 10 μM; 151-165 s: 20 μM. Each sensorgram is the average of at least three experiments. The decrease in Response Units (RU) after 165 s indicates analyte dissociation from the immobilized Bcl-2 upon HSBP2%D buffer injection. Control SPR experiments were carried out measuring sensorgrams on Bcl-xL and Mcl-1 (ligands) immobilized on COOH5 sensorchips as previously reported 40 at 25 °C in degassed HBSP2%D buffer. IS20, IS21 and ABT-199 (analytes) were injected at a flow rate = 30 μL/min, at the following concentrations: 0.3 μM (only for Mcl-1 experiments), 1 μM, 3 μM, 10 μM, 30 μM. Each sensorgram is the average of at least three experiments. The decrease in RU after 180 s indicates analyte dissociation from the immobilized ligands upon HSBP2%D buffer injection. Kinetic evaluation of the sensorgrams was carried out using the Qdat 4.0 program; best fittings were obtained with 2 sites and indicated in red (reported KD values refer to dissociation constants of the highest affinity site).

### *In vivo* experiments

Effect of IS21, ABT-199 and ABT-263 on tumor growth. All procedures involving mice and their care were authorized and certified by the decree no. 67/97A of the Italian Minister of Health and protocol 2560/97 of the Rome Health Service Unit (ASL RMB). Regina Elena National Cancer Institute approved all the procedures involving animals. 5 x 10^6^ U937 (in 50% Matrigel, Corning, NY, USA) or A375 cells were subcutaneously injected in the right flank of 6-8-week-old female immunodeficient athymic CD1 nude mice. IS21 was dissolved in 10% DMSO, 30% PEG400 and 60% NaCl (vehicle). Intraperitoneal treatment of vehicle or IS21 (8 mice for each group) every 24 h started when tumors reached the palpability (6 days for U937 and 7 days for A375 cells). ABT-199 and ABT-263 (Selleckchem, Houston, USA) were dissolved in 5% Tween80 (Acros Organics, New Jersey, USA), 10% DMSO (Sigma-Aldrich), 30% PEG400 (Sigma) and 55% NaCl (vehicle). Oral gavage of vehicle or ABT-199 or ABT-263 (4 mice for each group) every 24 h started when tumors reached the palpability. Animals were observed daily, weighted two times/week and tumor volume (mm^3^) calculated as length × width^2^ × π/6. At the end of the treatment the tumors were collected and weighed.

Pharmacokinetics. All details regarding pharmacokinetic analysis were reported in the Supplementary Information and in **Figure S8**.

### Statistical analysis

Experiments were replicated at least three times, unless otherwise indicated, and the data were expressed as average ± standard deviation (SD) or as average ± standard error of the mean (SEM). Differences between groups were analyzed with a two-sided paired or unpaired by Student's t-test. *In vivo* experiments were repeated twice. Differences between groups, analyzed with T-test, Wilcox o Mann-Withney, were considered to be statistically significant for p < 0.05.

## Results

### Identification of chemical scaffolds as new putative selective inhibitors towards anti-apoptotic Bcl-2 family members by ML guided VS

VS protocol was set up to select potential Bcl-2 inhibitors from more than 1 million compound commercial databases (https://vitasmlab.biz/ and https://chem-space.com/). From PubMed and ChEMBL databases all listed molecules assayed as ligand for Bcl-2 protein were retrieved with their associated bioaffinities (dataset) to build classification and regression quantitative structure-activity relationship (QSAR) models. The cleaned dataset was splitted into training and test sets to build predictive QSAR models. Based on the elaborations, among built soft voting classification models, those developed using the hybrid features (fingerprints and descriptors) revealed to be quite robust (**Tables S1-S7**). In particular for functional cell based (FA Class) assay, the best model showed a cross-validated accuracy level as high as 0.84 with a Matthew correlation coefficient (MCC) of 0.65, while for the biochemical based (BA Class) assay, the cross-validated accuracy and MCC were equal to 0.96 and 0.91, respectively. The developed models were then applied as filtering tools to rank the commercial small molecules database and prioritize the most promising compounds to be experimentally tested as potential Bcl-2 inhibitors. As FA and BA class of compounds derived from different biological data, it was decided to initially select the 30 highest ranked molecules by each model to a total of 60 top ranked candidate molecules. As three of them were not available from vendor, and 8 were filtered out because of precipitate formation when reconstituted in DMSO, we focused our attention on 49 compounds (**Table S8**).

### Analysis of the effect of a panel of putative inhibitors of anti-apoptotic Bcl-2 family members on cell viability of leukemia and melanoma cells

**Figure 1:**
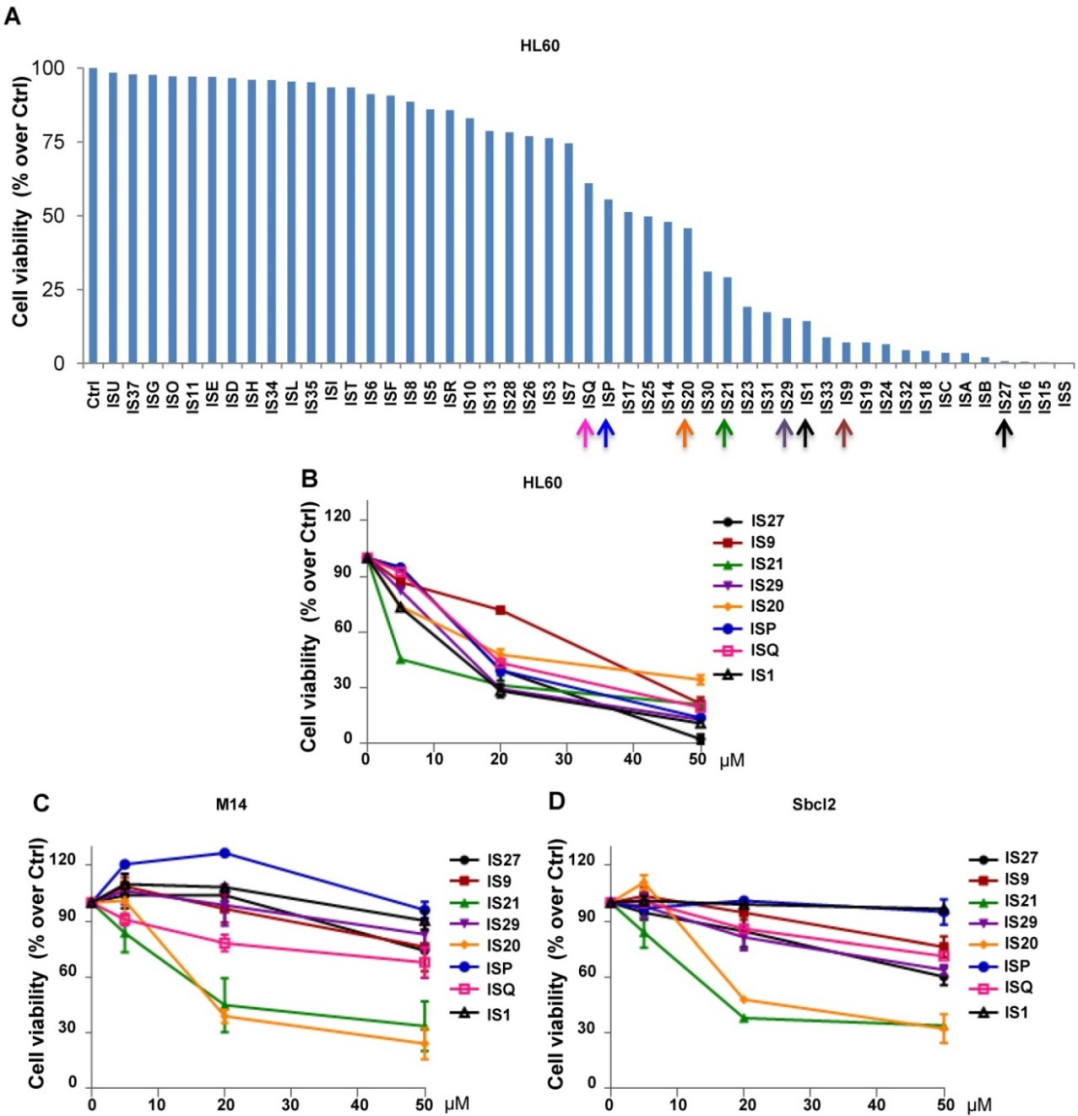
**Cytotoxic screening of putative inhibitors of Bcl-2 anti-apoptotic proteins**. (**A**) Flow cytometric quantification of propidium iodide exclusion assay in HL60 cells treated with the 49 putative Bcl-2 family inhibitors (50 μM, 72 h). (**B**) Analysis of cell viability by CellTiterGlo assay in HL60 cells treated for 24 h with concentrations ranging from 5 to 50 μM of the indicated compounds. (**C-D**) Analysis of cell viability by MTT assay in (**C**) M14 and (**D**) Sbcl2 melanoma cell lines treated for 72 h with concentrations ranging from 5 to 50 μM of the indicated compounds. (**A-D**) The results are reported as “viability of treated cells/viability of control cells (Ctrl)” × 100. Data are reported as mean of two (HL60) or three (M14 and Sbcl2) experiments ± SEM.

The 49 compounds were firstly evaluated at fixed concentration on the viability of HL60 cells. As reported in **Figure 1A,** HL60 cells were differently sensitive to the analyzed compounds in terms of cell viability. In particular, 22 compounds showed a reduction in cell viability ranging from 60 to 10% respect to control cells. We next investigated the effect of these 22 compounds on HL60 cells viability, performing a treatment with 5, 20 and 50 µM for 24 h (data not shown). The 8 compounds (IS1, IS9, IS20, IS21, IS27, IS29, ISP and ISQ, as sdf file format available in additional supporting files) inducing a dose-dependent effect in HL60, with IC_50_ values ranging from 10.9 µM to 34 µM (**Figure 1B),** were further tested in two human melanoma cell lines, M14 and Sbcl2. As reported in **Figure 1C-D,** both M14 and Sbcl2 melanoma cells displayed a different sensitivity to the 8 compounds, being IS20 (IC_50_ 19.8 µM and 21 µM, for M14 and Sbcl2, respectively) and IS21 (IC_50_ 16.7 µM and 19.8 µM, for M14 and Sbcl2, respectively) the most effective in reducing cell viability. Of note, these two compounds had no significant effect on normal human fibroblasts viability (data not shown).

### Binding of putative inhibitors of anti-apoptotic members of Bcl-2 family to Bcl-2, Bcl-xL and Mcl-1

**Figure 2:**
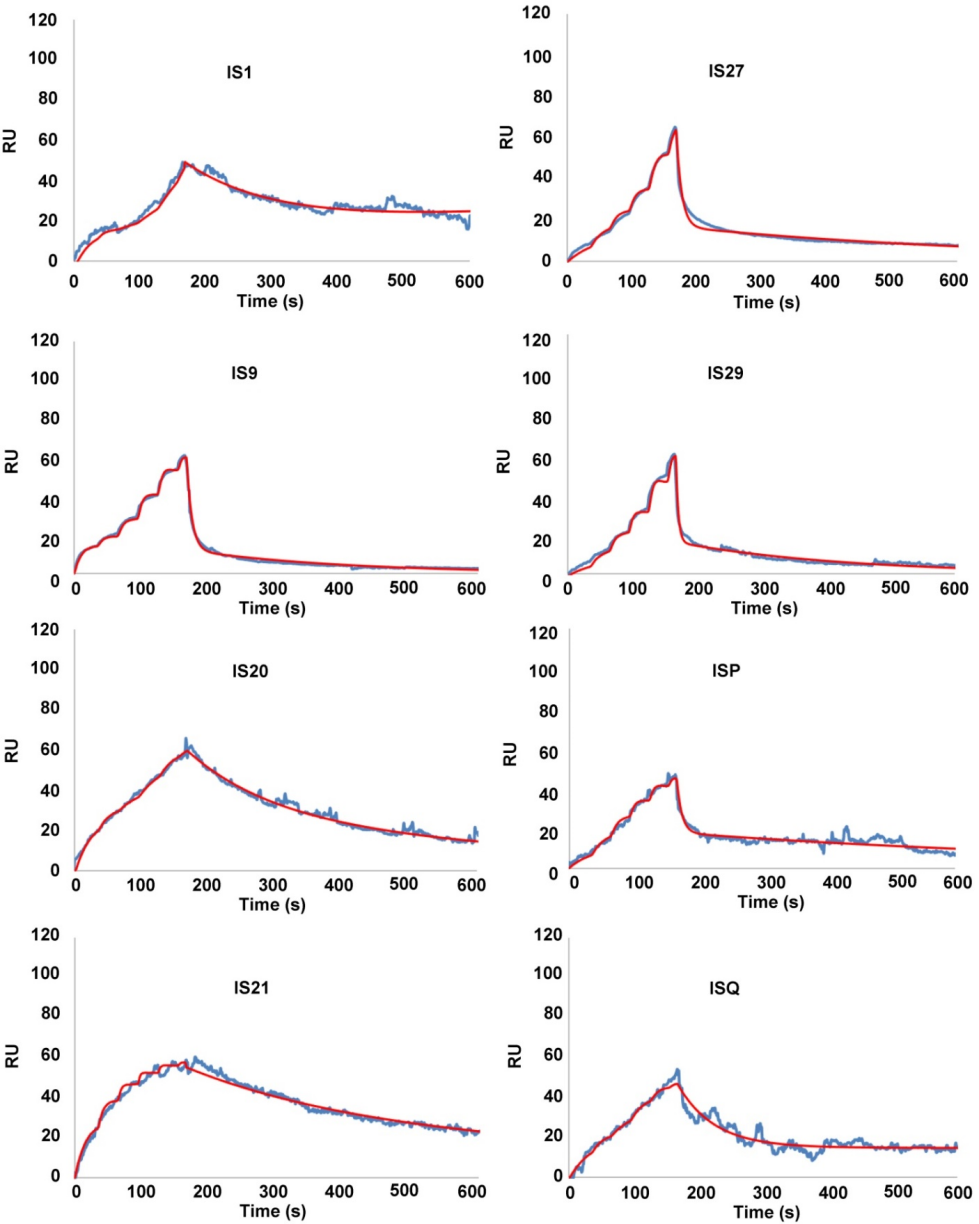
**SPR analysis of the interaction between putative inhibitors of Bcl-2 anti-apoptotic members and Bcl-2 protein**. Bcl-2 was immobilized *via* amine coupling onto a COOH5 sensorchip; Bcl-2 family inhibitors (analytes) were injected at a flow rate = 100 μL/min, at the following concentrations. 0-30 s: 0.625 μM; 31-60 s: 1.25 μM; 61-90 s: 2.5 μM; 91-120 s: 5 μM; 121-150 s: 10 μM; 151-165 s: 20 μM. The increase in Response Units (RU) relative to baseline indicates complex formation between the immobilized Bcl-2 ligand and the analytes upon Bcl-2 family inhibitors injection. The decrease in RU after 165 s indicates analyte dissociation from the immobilized Bcl-2 upon buffer injection. Full fittings with 2 sites are indicated in red. The fitted KD values are the following. IS1: 0.48 ± 0.11 µM; IS9: 4.0 ± 0.7 µM; IS20: 0.32 ± 0,09 µM; IS21: 0.19 ± 0.07 µM; IS27: 4.6 ± 0.9 µM; IS29: 3.4 ± 1.1 µM; ISP: 0.77 ± 0.14 µM; ISQ: 0.53 ± 0.1 µM.

**Figure 3:**
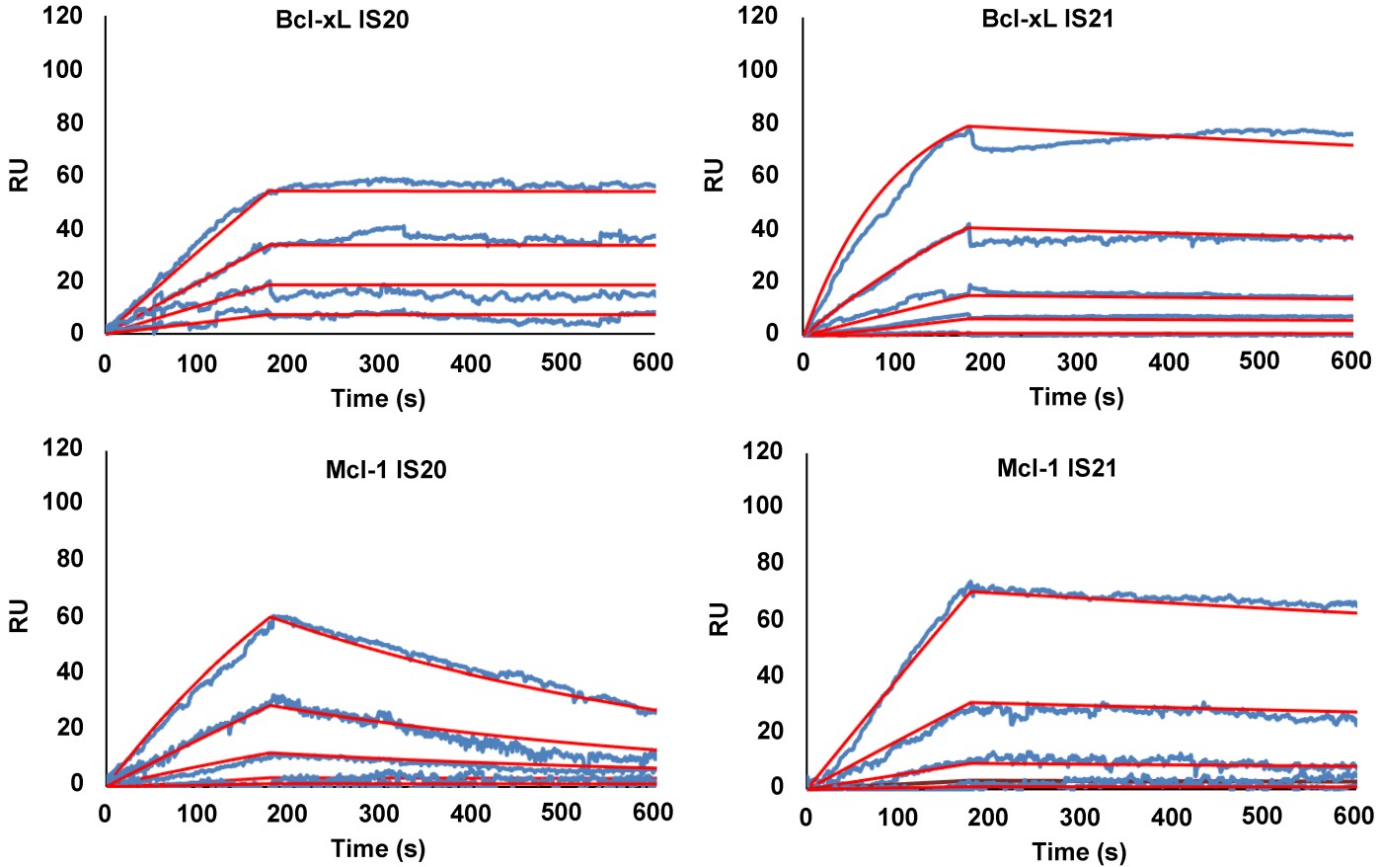
** SPR analysis of the interaction between IS20 or IS21 and Bcl-xL and Mcl-1 proteins**. SPR experiments were carried out measuring sensorgrams on Bcl-xL and Mcl-1 (ligands) immobilized on COOH5 sensorchips at 25 °C in degassed HBSP2%D buffer. IS20 and IS21 (analytes) were injected at a flow rate = 30 μL/min, at the following concentrations: 0.3 μM (only for Mcl-1 experiments); 1 μM; 3 μM; 10 μM; 30 μM. Each sensorgram is the average of at least three experiments. The decrease in RU (Response Units) after 180 s indicates analyte dissociation from the immobilized ligands upon HSBP2%D buffer injection. The fitted KD values for Bcl-xL are the following. IS20: 0.42 ± 0.09 μM and IS21: 0.51 ± 0.17 μM. The fitted KD values for Mcl-1are the following. IS20: 3.9 ± 0.30 μM and IS21: 1.16 ± 0.14 μM;

To eliminate false positive compounds, SPR experiments were performed to investigate the ability of the most promising 8 compounds, coming out from our *in silico* and cytotoxicity studies, to bind to Bcl-2 immobilized on COOH5 sensorchip. As reported in **Figure 2** and** Table 1**, affinities in the micromolar or in the submicromolar range were obtained for all tested compounds, and in particular for compounds IS20 and IS21. The fitted KD values were the following: IS21, 0.19 ± 0.07 µM; IS20, 0.32 ± 0,09 µM; IS1, 0.48 ± 0.11 µM; ISQ, 0.53 ± 0.10 µM; ISP, 0.77 ± 0.14 µM; IS29, 3.40 ± 1.1 µM; IS9, 4.0 ± 0.70 µM; IS27, 4.60 ± 0.90 µM. As a further control, a SPR experiment was also carried out with ABT-199 as analyte **(Figure S9)**. ABT-199 dissociation was very slow, thereby impairing a precise evaluation of the dissociation constant: however, a fitted KD value of 16 ± 11 nM was obtained. SPR experiments were also carried out using Bcl-xL and Mcl-1 as ligands and IS20, IS21 and ABT-199 as analytes. Affinities in the micromolar or in the submicromolar range were obtained for IS20 and IS21. The fitted KD values for Bcl-xL were the following: IS20, 0.42 ± 0.09 μM; IS21, 0.51 ± 0.17 μM; ABT-199, 1.40 ± 0.30 μM. The fitted KD values for Mcl-1 were the following: IS20, 3.90 ± 0.30 μM; IS21, 1.16 ± 0.14 μM; negligible interaction was observed for ABT-199 (**Figure 3**, **Table 1, Figure S9**). Starting from the KD analysis, the selectivity index of both compounds for Bcl-2, Bcl-xL and Mcl-1 proteins have been also calculated. In particular, IS21 showed to be about 2.7 and 6 times more selective for Bcl-2 than for Bcl-xL and Mcl-1, respectively. As regard IS20, it showed a similar selectivity profile between Bcl-2 and Bcl-xL, while it was 12 times more selective for Bcl-2 than Mcl-1.

### Analysis of apoptosis induced by IS20 and IS21 in melanoma, NSCLC and leukemia cells

**Figure 4:**
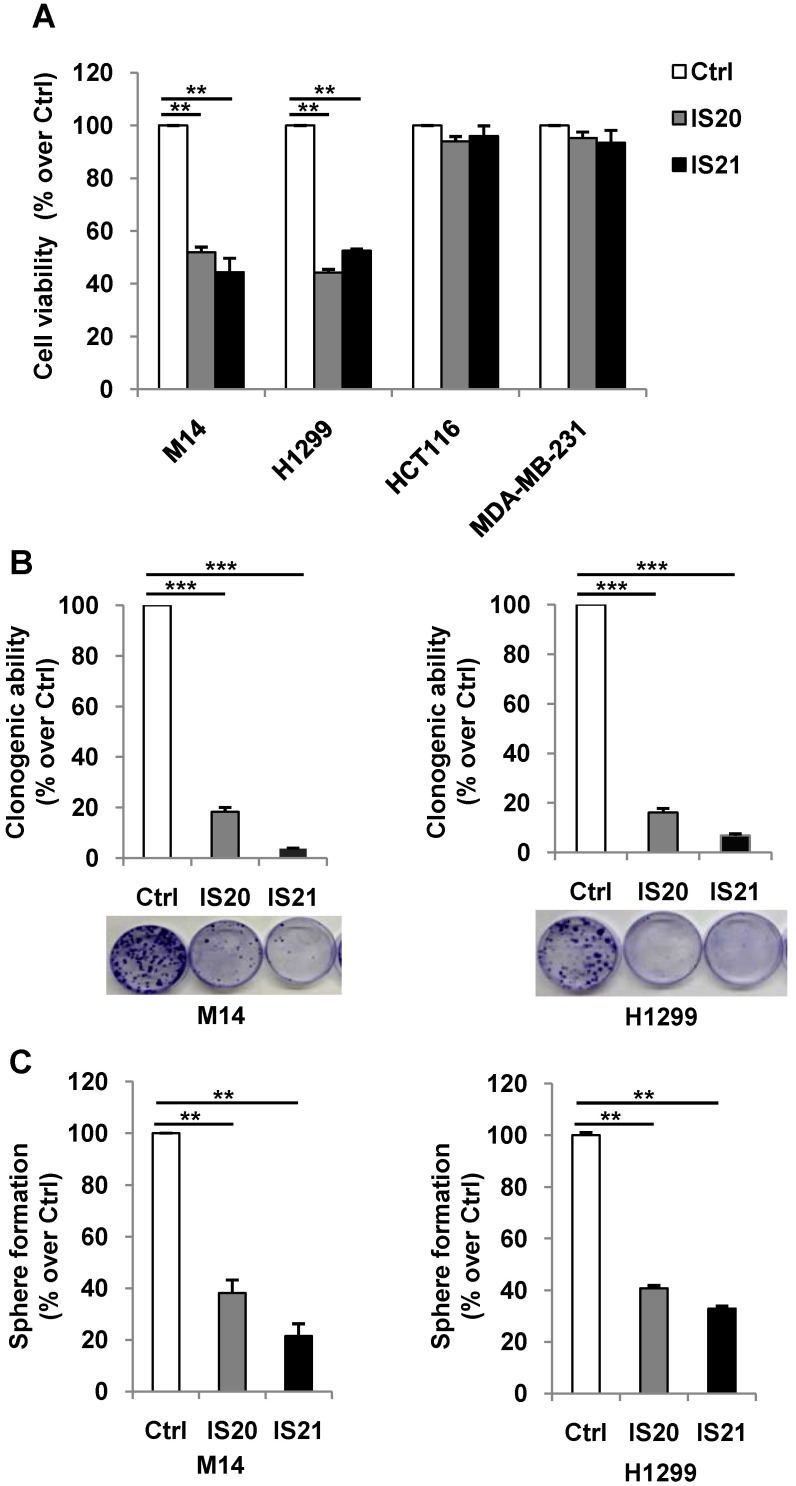
** Analysis of the effect of IS20 and IS21 on cell viability, clonogenic ability and tumor sphere formation in cancer cells. (A)** Analysis of cell viability by MTT assay in M14, H1299, HCT116 and MDA-MB-231 cell lines treated with IS20 or IS21 (20 μM for 72 h). The results are reported as “viability of treated cells/viability of control cells (Ctrl)” × 100. **(B)** Representative images and quantification of clonogenic ability evaluated in M14 and H1299 cells treated with IS20 or IS21 (20 μM for 72 h). Results are reported as percentage of clonogenicity of treated versus control cells. **(C)** Quantification of tumor sphere formation of M14 and H1299 cells treated with IS20 or IS21 (20 μM for 72 h). Results are reported as percentage of tumor sphere formation of treated versus control. **(A-C)** Data are reported as mean ± SD of three independent experiments. p-values were calculated between control (Ctrl) and treated cells, ** p < 0.001 and *** p < 0.0001.

**Figure 5:**
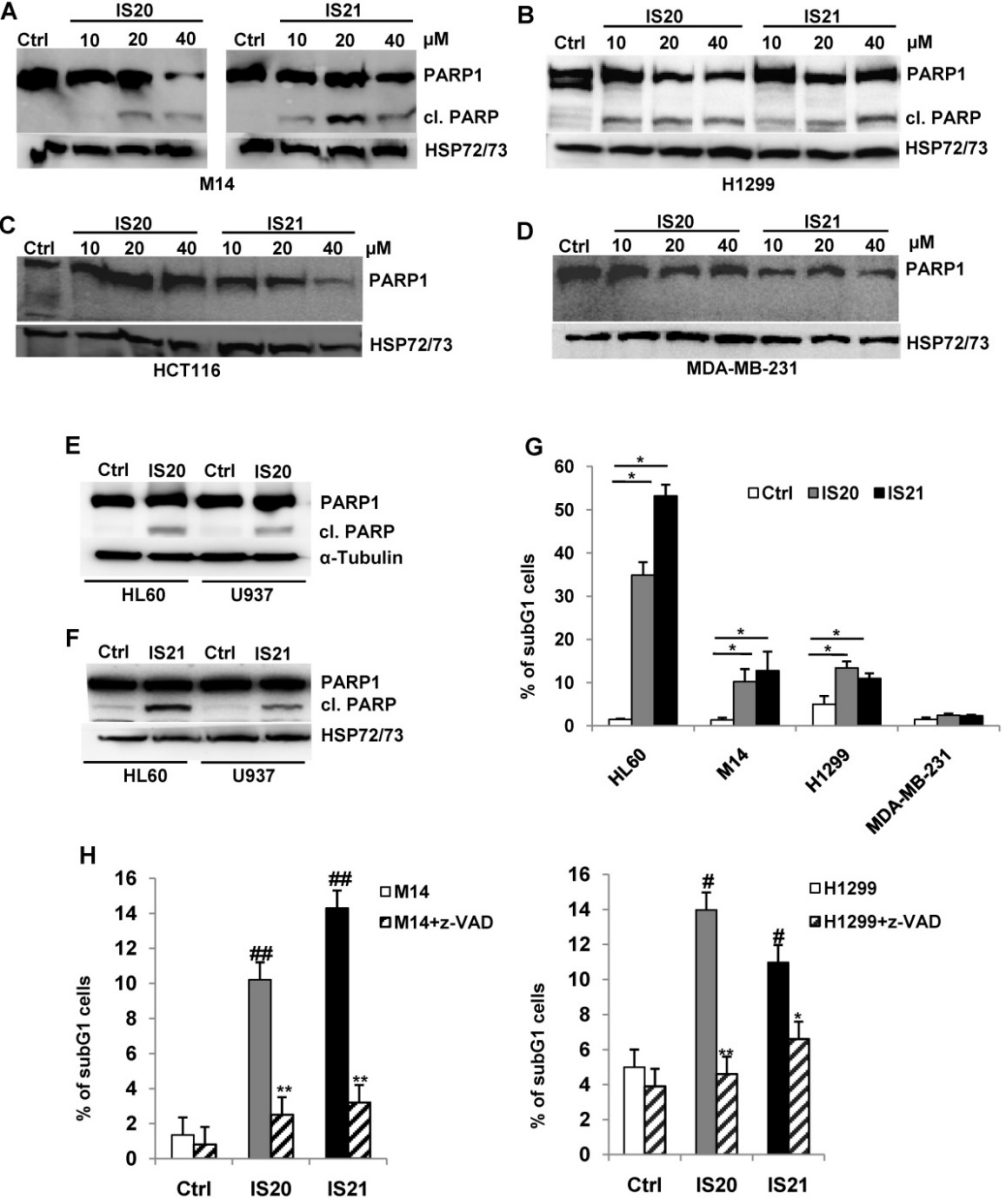
** Analysis of apoptosis induction by IS20 and IS21 in leukemia, melanoma and NSCLC cells.** (**A-D**) Western blot analysis of PARP1 cleavage (cl. PARP) in (**A**) M14, (**B**) H1299, (**C**) HCT116 and (**D**) MDA-MB-231 cell lines exposed to IS20 or IS21 at the indicated concentrations for 72 h. (**E-F**) Western blot analysis of PARP1 cleavage (cl. PARP) in HL60 and U937 cells exposed to IS20 or IS21 at 20 μM for 24 h. (**A-F**) Reported images are representative of two independent experiments with similar results. HSP72/73 or α-Tubulin are shown as loading and transferring control. (**G**) Flow cytometric quantification of cells in the subG1 peak of cell cycle distribution by propidium iodide staining in HL60 cell line treated with IS20 or IS21 (20 μM for 24 h) and in M14, H1299 and MDA-MB-231 cell lines treated with IS20 or IS21 (20 μM for 72 h). Data are reported as mean ± SD of three independent experiments. p-values were calculated between control (Ctrl) and treated cells, * p < 0.05. (**H**) Flow cytometric quantification of cells in the subG1 peak by propidium iodide staining in M14 and H1299 cell lines treated with IS20 or IS21 (20 μM, 72 h) in presence or absence of the pan caspase inhibitor z-VAD (50 μM). p-values were calculated between treated control (Ctrl) cells, # p < 0.01, ## p < 0.001 or between cells treated with IS20 or IS21 alone or in combination with z-VAD, * p < 0.05, ** p < 0.005. (G-H) Data are reported as mean of two (H) or three (G) experiments ± SD.

We further investigated the biological effect of IS20 and IS21 in four human cancer cell lines, representative of different solid tumor histotypes and expressing different levels of anti-apoptotic proteins (**Figure S9**). In particular, M14 melanoma, H1299 NSCLC, HCT116 colon and MDA-MB-231 breast carcinoma cells were used. As shown in **Figure 4A,** M14 and H1299 cells showed a significant inhibition of cell viability after exposure to both compounds, with respect to controls. On the contrary, HCT116 and MDA-MB-231 cell viability was not significantly perturbed by the treatment. Consistent with these results, after IS20 or IS21 exposure both the colony and sphere formation capacity was impaired in M14 and H1299 cell lines, when compared to control cells (**Figure 4B-C**). In particular, IS20 or IS21 at 20 μM induced a significant decrease of about 80-90% cell colony formation and about 60% of sphere formation capacity, in M14 and H1299 treated cells respect to controls. Notably, reduction of cell viability was accompanied by the cleavage of PARP1 evaluated by western blotting (**Figure 5A-B**), not evident in HCT116 or MDA-MB-231 treated cells (**Figure 5C-D**)**.** Western blotting analysis also demonstrated the cleavage of PARP1 in HL60 and U937 cells after treatment with both IS20 and IS21 (**Figure 5E-F**). In addition, flow cytometric analysis showed the presence of subG1 peak of cell cycle distribution after treatment of HL60, M14 and H1299 cells with the two compounds, while subG1 peak was not evident in MDA-MB-231 cell line (**Figure 5G**). To confirm the ability of IS20 and IS21 to induce apoptosis, we treated M14 and H1299 cells with both compounds in the presence of the pan-caspase apoptosis inhibitor, z-VAD. As reported in **Figure 5H,** z-VAD significantly decreased the percentage of cells in subG1 peak in both cell lines, demonstrating that the observed subG1 peak and PARP1 cleavage were due to apoptosis activation.

### Analysis of autophagy induced by IS20 and IS21 in both melanoma and NSCLC cells

**Figure 6:**
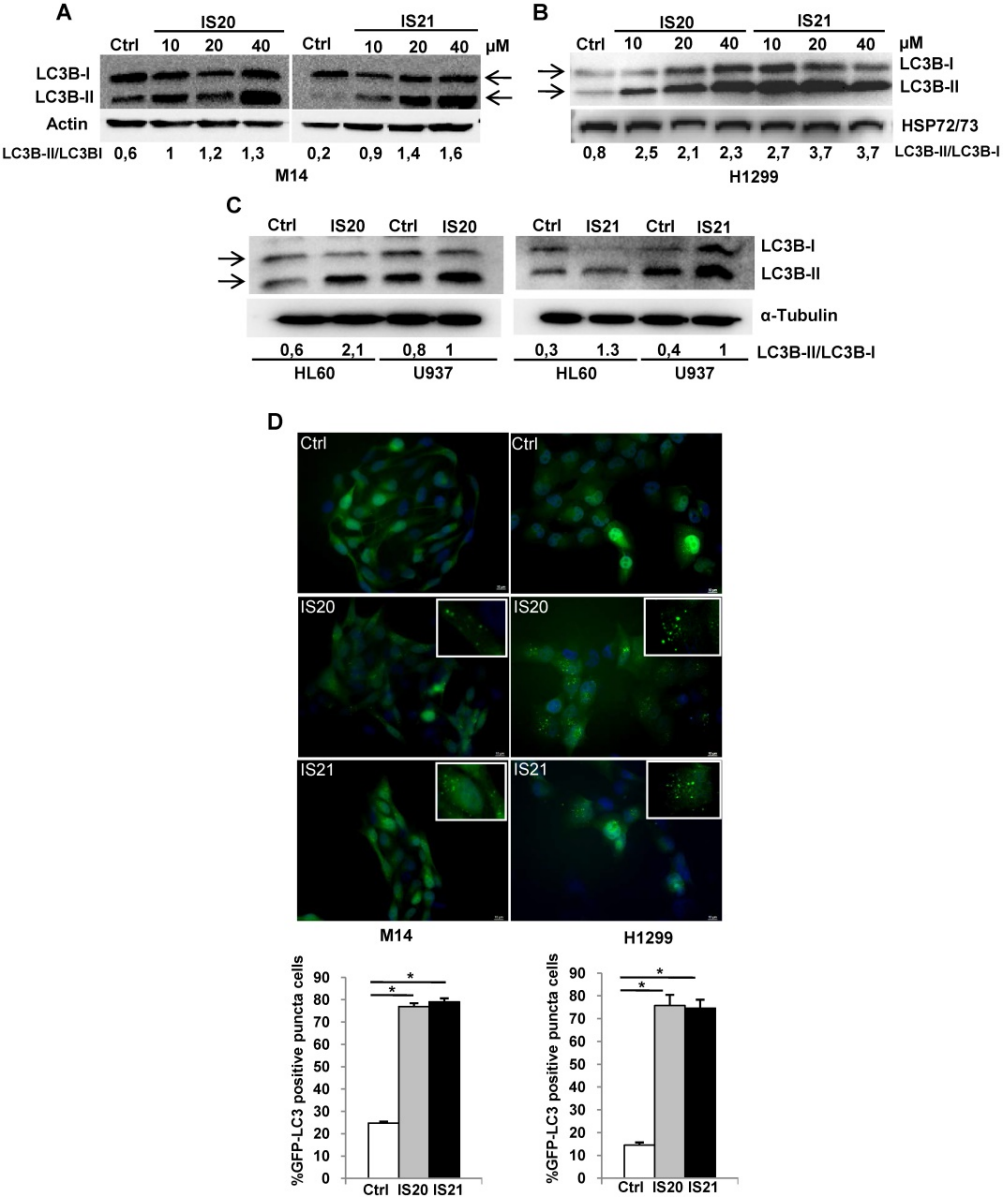
** Analysis of autophagosome accumulation in M14 and H1299 cells after treatment with IS20 or IS21.** Western blot analysis of LC3B-I and LC3B-II protein levels in (**A**) M14, and (**B**) H1299 cell lines untreated (Ctrl) or treated with IS20 or IS21 for 72 h at the indicated concentrations. (**C**) Western blot analysis of LC3B-I and LC3B-II levels in HL60 and U937 cell lines untreated (Ctrl) or treated with IS20 or IS21 (20 μM for 24 h). (**A-C**) Reported images are representative of two independent experiments with similar results. HSP72/73, Actin or α-Tubulin are shown as loading and transferring control. The arrows indicate LC3B form I and LC3B form II. Ratio of LC3B-II on LC3B-I levels, after normalization on HSP72/73, Actin or α-Tubulin, is quantified by densitometric analysis. (**D**) (Upper panels) Representative images and (lower panels) relative quantification of fluorescence microscopic analysis of LC3B puncta positive cells in M14 EGFP-LC3B and H1299 EGFP-LC3B transfected cells, treated with IS20 or IS21 (20 µM for 72h). The results are reported as “percentage of EGFP-LC3B positive cells with dots”. Cells with more than 10 puncta were considered autophagy-positive. p-values were calculated between untreated (Ctrl) and treated cells, * p < 0.05.

**Figure 7:**
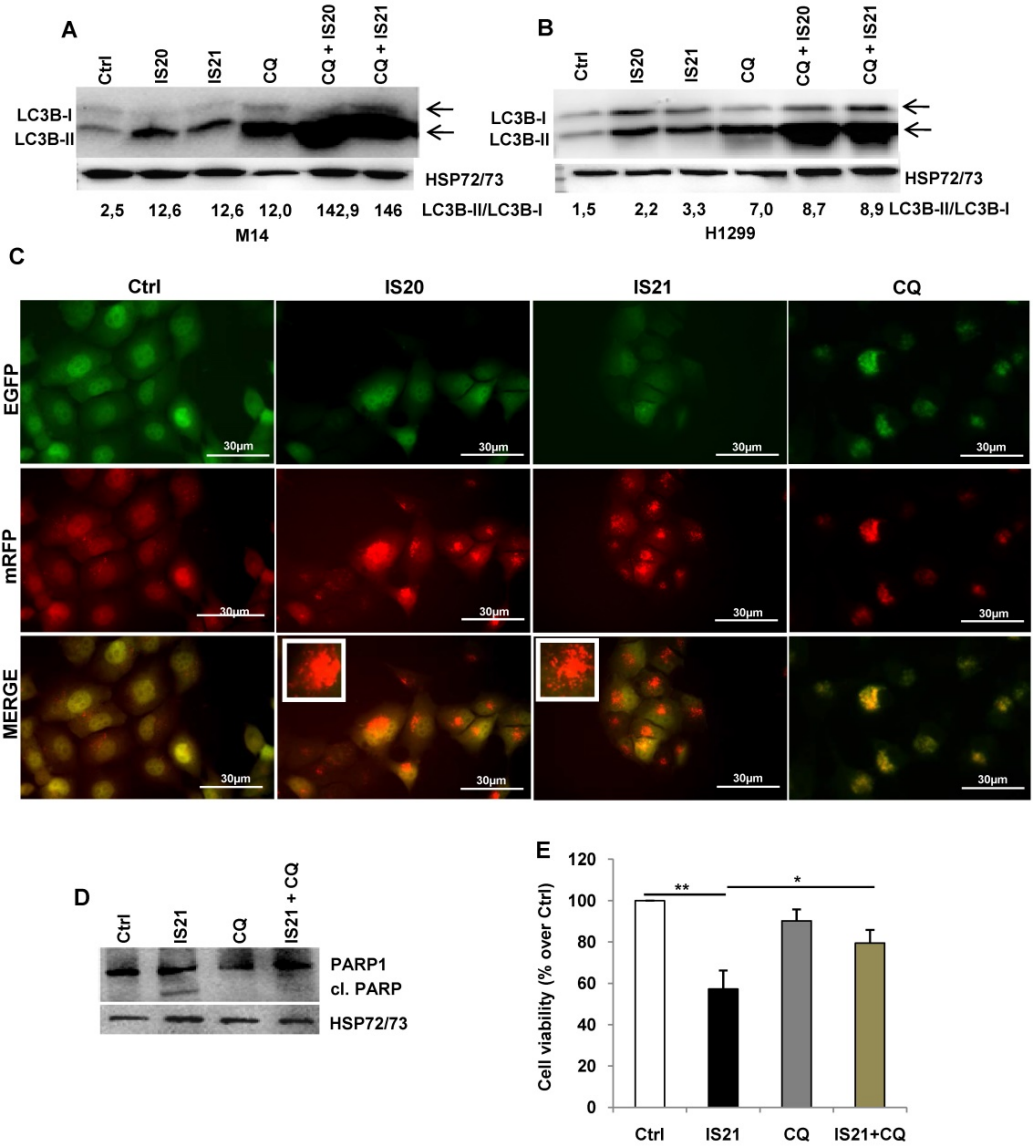
** Investigation of the role of the autophagy process induced by IS20 and IS21 in the promotion of apoptosis in M14 and H1299 cells.** Western blot analysis of LC3B-I and LC3-II levels in (**A**) M14 and (**B**) H1299 cells, untreated (Ctrl) and treated with IS20 or IS21 (20 µM) and cloroquine (CQ, 5 µM) alone or in combination for 72 h. Densitometric analysis of the ratio between LC3B-II and LC3B-I, after normalization on HSP72/73, were reported. (**A-B**) The arrows indicate LC3B form I and LC3B form II. (**C**) Representative images of fluorescence microscopy analysis of yellow- and red-fluorescence EGFP-LC3B punctate vesicular structures, indicative of autophagosomes and autolysosomes accumulation, respectively, in H1299-ptf-LC3 cells treated with IS20 (20 µM), IS21 (20 µM), or with CQ (5 µM) for 72 h. (**D**) Western blot analysis of PARP1 cleavage (cl. PARP), in M14 cells untreated (Ctrl) or treated with IS21 (20 µM, 72 h), alone or in combination with CQ (5 µM). (**E**) Analysis of cell viability by MTT assay in M14 cells untreated (Ctrl) or treated with IS21 (20 µM, 72 h), alone or in combination with CQ (5 µM). The results are reported as “viability of treated cells/viability of control cells (Ctrl)” × 100. Data are reported as mean ± SD of three independent experiments. * p < 0.05 and ** p < 0.01. (**A,B,D**) Reported images are representative of two independent experiments with similar results. HSP72/73 is shown as loading and transferring control.

Since Bcl-2 protein has been reported to regulate the autophagic process by interaction with Beclin 1 37, we next investigated whether IS20 and IS21 could affect *in vitro* autophagy. To monitor the activation of autophagic flux, we evaluated the expression of LC3B form II, which is an autophagosome-associating form (LC3B-II, 16 kDa) and LC3B form I (LC3B-I, 18 kDa), and calculated the ratio between the two forms. We observed higher level of the ratio of LC3B-II on LC3B-I protein levels upon treatment of M14 and H1299 cells with increasing concentration of IS20 and IS21 (**Figure 6A-B**). Increased ratio of LC3B-II on LC3B-I has been also reported in both HL60 and U937 treated cells (**Figure 6C**). In order to visualize autophagosomes and quantify the accumulation of the membrane-bound form of LC3B-II on autophagic vesicles, M14 and H1299 cells stably transfected with EGFP-LC3B fusion protein were exposed to IS20 or IS21. By fluorescence microscopy experiments, we observed an enhancement of LC3B punctate structure formation, indicative of autophagosome accumulation after treatment of both cell lines, when compared to control cells (**Figure 6D**). To determine whether the higher ratio of LC3B-II/LC3B-I observed in both IS20 and IS21 treated cells was generated by increased *de novo* autophagosome biosynthesis or by late-state autophagy inhibition, we treated the cells with IS20 or IS21 in the presence or absence of CQ, an inhibitor of late stage of autophagy, and evaluated the ratio of LC3B form II on LC3B form I. As reported by western blot analysis, and as expected, CQ treatment caused an increase of LC3B-II/I ratio. This increment was more evident when M14 and H1299 cells were exposed to the combination of IS20/CQ or IS21/CQ (**Figure 7A-B**), indicating that both IS20 and IS21 activate autophagy in these cells. To confirm the induction of a complete autophagic flux and to determine the autophagosome maturation and autolysosome formation we used a pH-sensitive, double-tagged mRFP-GFP-LC3B reporter (ptf-LC3) in H1299 cells. As reported in **Figure 7C**, H1299 cells expressing the ptf-LC3 construct and treated with IS20 or IS21 showed increased red fluorescence, indicating that both compounds induced autophagy and led to the correct fusion between autophagosomes and lysosomes to yield autolysosomes. On the contrary, as expected, CQ determined an increased yellow fluorescence, indicating a block in autolysosomes formation.

As the functional crosstalk between autophagy and apoptosis has been reported 41, we investigated whether induction of autophagy by IS21 was an independent or apoptosis-linked process, evaluating the cleavage of PARP1 in M14 cells treated with IS21 alone or in combination with CQ. As shown in **Figure 7D**, the cleavage of PARP1 was observed only in the M14 cells treated with IS21, while it was inhibited by CQ, thus demonstrating that the autophagy process promotes apoptosis induction observed in IS21 treated cells. In accordance with these results, we observed a significant recovery of M14 cell viability after treatment of cells with IS21 in combination with CQ (**Figure 7E**).

The ability of IS20 and IS21 to affect cell viability, colony and sphere formation, to promote cleavage of PARP1, and to increase the expression of LC3B-II was also confirmed on A375 melanoma cell line (**Figure S10**).

The effect of ABT-199 and ABT-263 on cell viability, apoptosis and autophagy of M14 cells was also evaluated. As reported in **Figure S11**, the two compounds induced a reduction of cell viability similar to that observed after treatment with IS21. They also determined PARP1 cleavage and increased the expression of LC3B-II protein.

### Binding mode analysis of inhibitors of anti-apoptotic members of Bcl-2 family

**Figure 8:**
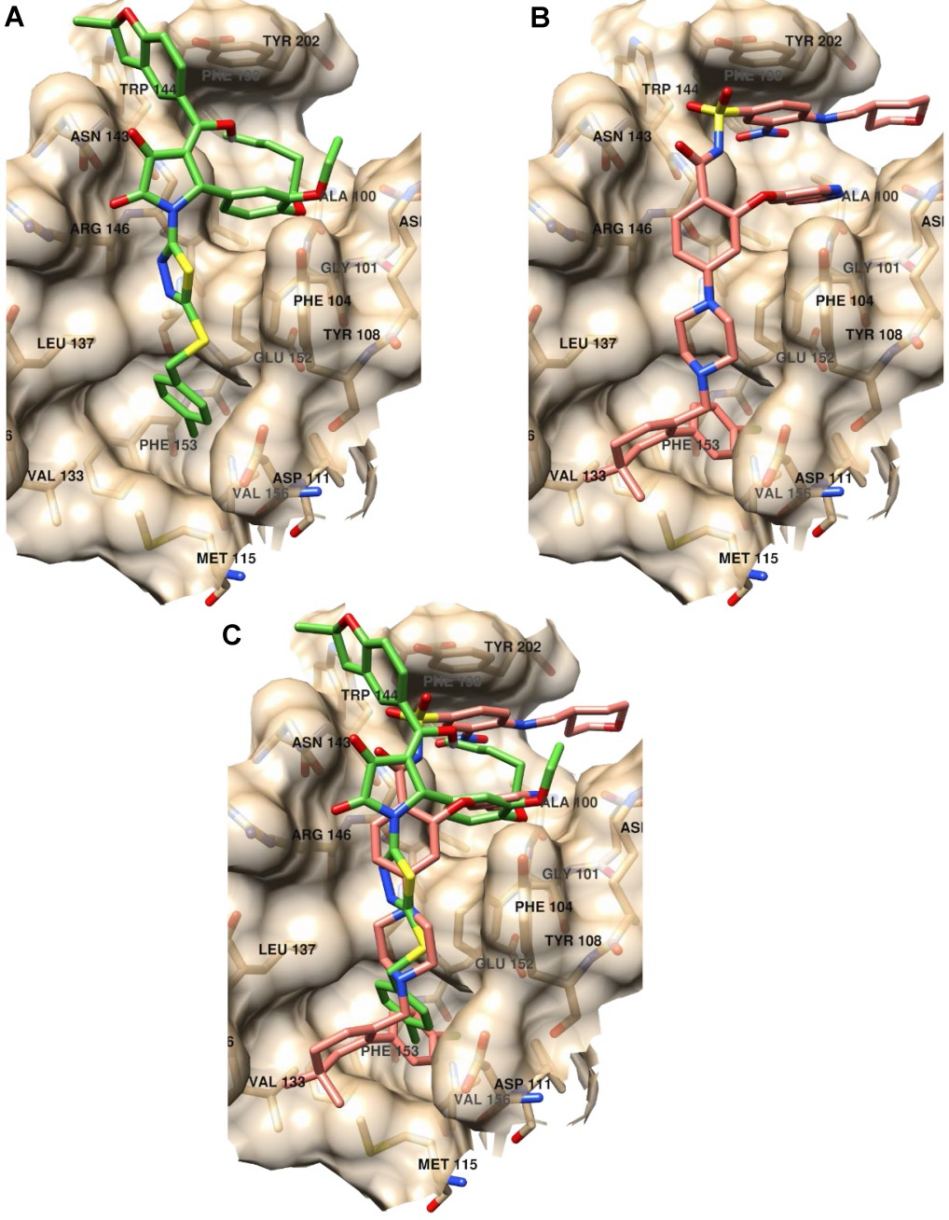
**Binding mode analysis of IS21 and ABT-199**. (**A**) IS21 (green colored carbon atoms), (**B**) ABT-199 (orange colored carbon atoms) and (**C**) overlapped IS21/ABT-199 docked conformation in the *wild type* Bcl-2 protein (pdb id 6O0K, gold colored surface and carbon atoms).

Further, we analyzed the binding modes of IS20 and IS21 by means of molecular docking simulation. The docking energies, calculated by the VINA scoring function available in Smina software compared with the KD_1_, showed a Pearson correlation coefficient of 0.85 indicating the VINA scoring function quite suitable to predict Bcl-2 inhibitor potencies. In order to avoid any redundancy, herein only details on the putative binding mode of IS21 are reported. Investigation on the IS21 binding mode, revealed IS21 ability to bind into the *wild type* and mutated Bcl-2 protein, with an almost fixed conformation (**Figure S1**) that somehow recalled those of ABT-199, which, showed a unique binding conformation in the experimental co-crystallized complexes (**Figure S1**). Although the great structural difference from ABT-199, the IS21 docked conformation fully overlapped the ABT-199 bound conformation, being almost able to replicate all main ligand/protein interactions (**Figure 8**), and to place some new interactions in the benzofurane moiety (**Figure S1 and S3**).

In particular, the 4-clorophenyl group of IS21 occupied the same pocket of the corresponding 4-clorophenyl moiety of ABT-199 formed by residue Met115, Val156, Glu152, Phe112, Ala149, Asp111, Phe3153 and Phe104, where the Beclin 1 BH3 domain Leu116 side chain is normally bound, as observed in the co-crystallized Bcl-2/Beclin 1 complex (pdb entry code 5VAX) 42. The thiadiazolylpyrrolidindione intermediate IS21 group partially mimed four residues of the bound Beclin BH3 α-helix (Val118, Thr119, Gly120 and Asp121) placing hydrophobic interactions with residues Asp111, Tyr108, Phe104, Leu137, Gly145 and two strong hydrogen bonds with the two pyrrolidindione carbonyl oxygens and the Asn143-N1 (2.77Å) and Arg146-Nω (3.02Å), respectively (**Figure S2**). In comparison, ABT-199 also mimicked the same four residue of the BH3 α-helix with the 4-pyperazinylbenzamide group making just one only weak hydrogen bond between the benzamide oxygen and Asn143-N1 (3.36Å). IS21 fulfilled a wide and shallow Bcl-2 pocket with the catechol group and the pentyl and the ethyl chain linked to its oxygens. In a similar fashion, ABT-199 made a large amount of hydrophobic interactions in the same pocket (Phe104, Arg107, Asp103, Ala10, Val148, Phe198 and Tyr202) fulfilled with the pyrrolopyridine and 4-amino-3-nitrobenzensulphonamide groups. Interestingly, for the BH3 domain, only the Phe123 side chain was found to be in that large pocket (**Figure S3**). Moreover, IS21 docked conformation, differently from either ABT-199 or BH3 α-helix, made a further interaction with the benzofurane moiety, which filled a small Bcl-2 pocket formed by Asn143, Trp144 and Tyr202 residues.

To some extent, the predicted binding affinities for IS20 and IS21 could also give information of the ligands' propensity to bind the mutated Bcl-2 protein. In particular, based on the associated calculated docking energies, the binding of IS21 would not be affected by the G101V Bcl-2 mutation, reported to be related to resistance to ABT-199 28. Nonetheless, IS20 docked conformation did not show a unique binding mode. Docked conformation of IS20 into *wild type* Bcl-2 protein (**Figure S4**), compared to IS21 (**Figure S3**), displayed a different putative binding conformation, although it differed for one methylene group (the SAR paradox). This could reflect the lower affinity of IS20 respect to IS21 for Bcl-2 binding, evinced in SPR experiments. Further molecular docking experiments were carried out on Bcl-2, Bcl-xL and Mcl-1 proteins. In particular, as ABT-263 was reported to experimentally bind the three Bcl-2 family proteins 43, 44 we focused on ABT-263 containing complexes. From a survey on PDB, ABT-263 was experimentally co-cristallyzed with Bcl-2 and Bcl-xL *wild type* proteins (PDB Ids 6QGH and 4QNQ, respectively) while no complex with Mcl-1 was available. For the latter and for comparison purposes, a complex of Mcl-1 (PDB Id 6YBL) was selected by visually inspecting the co-crystallized ligand in all available Mcl-1 complexes. As Bcl-2, Bcl-xL and Mcl-1 proteins were also reported co-crystallized with the BH3 α-helix (PDB Ids 4BAS, 4QVF and 6QFI for Bcl-2, Bcl-xL and Mcl-1, respectively), molecular docking was further run into the separated protein conformations from those complexes. The IS21 docked poses into Bcl-2, Bcl-xL and Mcl-1 (**Figures S5-7**) indicate its ability to flexibly adapt to the different protein conformations in agreement with the SPR experiments that found IS21 ability to bind the three Bcl-2 family proteins. Comparison of the docked energies were also found in good agreement, as the VINA scoring function clearly indicated IS21 ability to bind better Bcl-2 and Bcl-xL proteins than Mcl-1 (**Table S9**) with a profile similar to that of ABT-263 43. In particular, the VINA scoring function correctly predicted IS20 and IS21 ability to bind Bcl-2 and Bcl-xL with comparable affinities, and more potently than Mcl-1 (**Table S9**).

Regarding the ADMET (Adsorption, Distribution, Metabolism, Excretion) properties, IS20 and IS21 were subjected to the SwissADME web tool to evaluate their DMPK (Drug, Metabolism, Pharmacokinetics) profiles in comparison with that of ABT-199. In general, IS21 showed an *in silico* ADMET profile with a slightly more favorable features profile respect to that of ABT-199 (**Table S10**).

### Pharmacokinetic analysis of IS20 and IS21, and effect of IS21 on *in vivo* growth of leukemia and melanoma models

The *in vivo* biopharmaceutical profile of IS20 and IS21 was analyzed by measuring their concentrations in plasma samples, obtained at fixed times after intraperitoneal administration of 100 mg/kg in healthy mice. Results showed that IS21 absorption was more rapid when compared to IS20, reaching maximum plasma concentrations 4 h after injection while maximum concentration was reached after 16 h for IS20 (**Figure S8A**). Unfortunately, we failed to accurately calculate the IS20 and IS21 pharmacokinetic parameters, because their concentrations in plasma remained high after 24 h of injection, probably due to the high dose used. Since IS21 showed a better pharmacokinetic profile than IS20, we measured pharmacokinetic parameters in plasma samples of mice intraperitoneally treated with 50 mg/kg IS21. Results showed that IS21 absorption reached maximum plasma concentrations 2 h after injection and that mean C_max_ was 4.1 µM, mean AUC_tot_ was 40.9 µM* per* h, mean t½ was 11 h, while mean total clearance was 26.3 mL/min/kg (**Figure S8B**).

**Figure 9:**
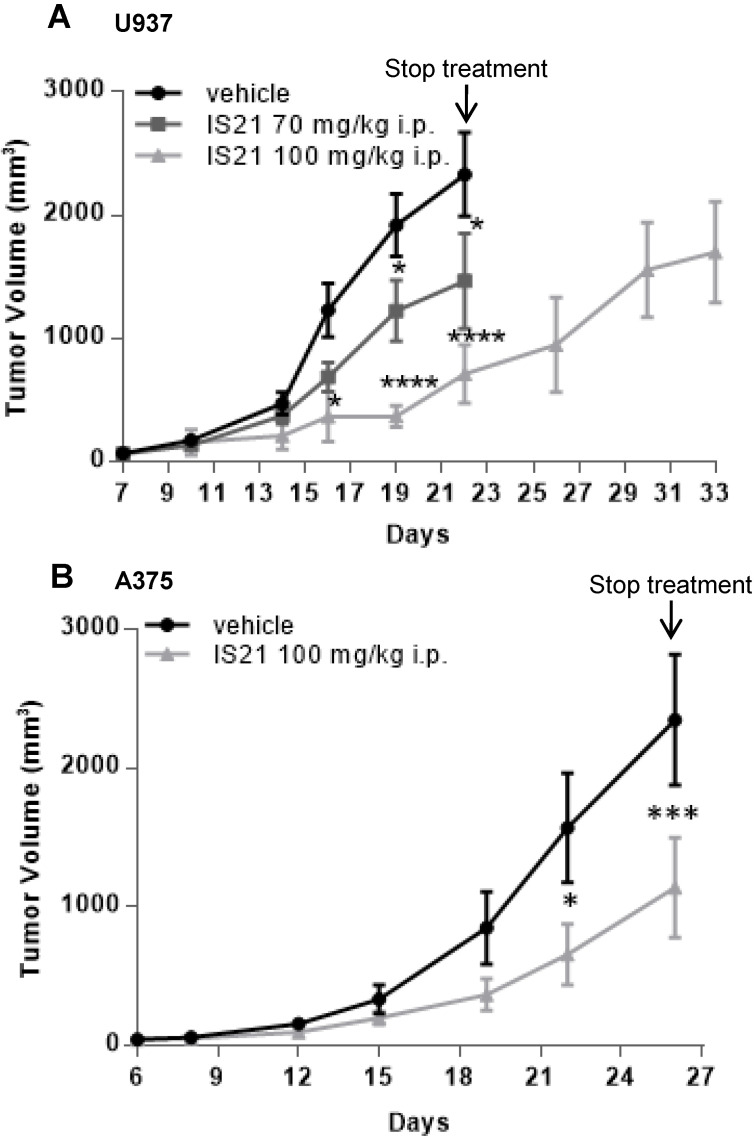
**Analysis *in vivo* of the effect of IS21 on both leukemia and melanoma models.** Analysis of *in vivo* tumor growth in nude mice injected with (**A**) U937 and (**B**) A375 cells and treated with vehicle or with IS21 at the indicated concentrations for (**A**) two or (**B**) three weeks. Experiments were repeated twice. * p < 0.05, *** p < 0.0002, **** p < 0.0001.

Based on pharmacokinetic analysis, we next evaluated the effect of IS21 on *in vivo* tumor growth of leukemic and melanoma derived xenografts. To this aim, U937 were injected subcutaneously and treated for two weeks with vehicle or with IS21 (70 mg/kg or 100 mg/kg, 5 days/week). As reported in **Figure 9A**, IS21 reduced significantly the growth of U937 xenografts starting from 16 and 19 days after cell injection for 100 mg/kg and 70 mg/kg, respectively. Importantly, mice treated with 100 mg/kg IS21, until day 22 showed a delay of tumor growth of about 11 days compared with control or those treated with IS21 70 mg/kg (**Figure 9A**).

The *in vivo* antitumor effect of IS21 was also confirmed in melanoma. In particular, mice injected with A375 melanoma cells were treated with vehicle or with IS21 (100 mg/kg, 5 days/week) for three weeks. As evident in **Figure 9B**, IS21 strongly reduced the *in vivo* tumor growth starting from 22 days after cell injection. IS21 treatment was highly tolerated, as no significant weight loss, diet consumption, and postural and behavioral changes were observed.

The effect of ABT-199 and ABT-263 on A375 *in vivo* tumor growth was also investigated and compared to the effect of IS21 (100 mg/kg, 5 days/week for two weeks). As reported in **Figure S12**, ABT-199, ABT-263 and IS21 showed a superimposable effect on tumor growth, being observed a reduction of xenografts ranging from 45% to 50% at day 22 after cells injection.

## Discussion

In this study, a ML guide VS approach was applied to select novel inhibitors of anti-apoptotic members of Bcl-2 family. We provided the evidence of *in vitro* pro-apoptotic and pro-autophagic effects of IS20 and IS21, two new inhibitors, in both hematological and solid tumor models from different origin. Moreover, we demonstrated an *in vivo* anti-tumoral activity of IS21 in both hematological and melanoma models. In particular, we firstly screened the sensitivity of leukemia cells to 49 selected molecules. Eight out of the 49 compounds, reducing cell viability from 40 to 90% and showing a dose-dependent effect respect to control cells, were chosen for testing in melanoma cells, finally pointing IS20 and IS21, showing higher anti-proliferative effect, without affecting viability of normal fibroblasts. SPR experiments performed on IS20 and IS21 demonstrated their affinity not only for Bcl-2 but also for Bcl-xL and Mcl-1 proteins. IS20 and IS21 showed cytotoxic effect, not only in leukemia and melanoma cells, but also in NSCLC cells, while they were ineffective in breast and colon cancer cells. Several factors could explain the different response of tumor cells to treatment, including not only basal expression levels of anti-apoptotic proteins, but also cell of origin of the tumor and the oncogenes driving malignant transformation associated to the histotypes 45. To this regard, we observed that melanoma as well as NSCLC cells, having relative higher levels of endogenous Bcl-2 and Mcl-1 proteins, respect to breast and colon cancer cells, were more sensitive to treatment. Dissecting the role of factors involved in the response to therapy will be helpful to the rational design of combination therapies, thus improving responses in patients with malignant disease. To this regard, inadequate patient selection was recognized as a limiting factor to reach better results in the oblimersen/dacarbazine clinical trials in patients with advanced melanoma 19. In the evaluation of BH3 mimetic drugs, other factors contribute to cellular responsiveness. For example, sensitivity to BH3-mimetic drugs correlate with the ratio between the pro-survival Bcl-2 family members and the pro-apoptotic BH3-only proteins 45. Moreover, the concept of cancer differential “addiction” to anti-apoptotic Bcl-2, Bcl-xL or Mcl-1 proteins were introduced to explain different survival of tumor cells depending of the expression levels of these proteins 45, 46. Multiple pro-survival Bcl-2 family members often regulate the survival of solid tumors, although this is not a generalized rule 45. In this context, it was reported that high Bcl-2 expression predicted sensitivity to ABT-199 in Non-Hodgkin lymphoma cell lines 25 and small cell lung cancer (SCLC) 29, and that SCLC and NSCLC cell lines expressing relatively higher levels of Bcl-2 were more sensitive to the putative Bcl-2 inhibitor, BDA-366 47, 48.

The use of IS20 or IS21 is conceivable as single agents or in combination with other chemotherapeutic drugs, taking into consideration tumor type, oncogenic drivers, and tumor heterogeneity 49-52. Several clinical trials are ongoing to evaluate the efficacy of Bcl-2 family anti-apoptotic inhibitors in solid tumors. These trials include the study of ABT-263 in combination with Dabrafenib and Trametinib for the treatment of patients with BRAF mutant melanoma or advanced solid tumors (NCT01989585), and the study of ABT-199 in combination with chemotherapy or pembrolizumab in patients with lung carcinoma (ClinicalTrials.gov Identifiers: NCT04274907, NCT04422210). It is conceivable that the use of Bcl-2 family inhibitors, in combination with other drugs, could allow the use of lower doses of both drugs employed in the combination, with consequent less toxicity and reduction of trombocytopenia, which is frequently associated with ABT-263 treatment 23, 24.

Drug sensitivity of melanoma and NSCLC cells to IS20 and IS21 was also confirmed by colony assay and 3D culture. The reduction of tumor sphere formation observed after treatment with both IS20 and IS21 suggested that these compounds could be also successful in eradicating cancer stem cells (CSC) component by targeting Bcl-2 family protein. In fact, sphere formation ability also represents an *in vitro* method commonly used to identify CSC and study their properties 53. Different mechanisms could be responsible of resistance to treatment in CSC, including Bcl-2-dependent chemoresistance 54. In addition to driving response to therapy, CSC have shown to play a role in tumor growth, local invasion, and distant metastasis 55. In line with this evidence, we recently demonstrated that Bcl-2 and Bcl-xL play essential role in the maintenance of CSC phenotype in melanoma 11.

Proteolytic cleavage of PARP1, subG1 peak and the pan-caspase inhibitor z-VAD demonstrated the ability of IS20 and IS21 to induce apoptosis in leukemia, melanoma and NSCLC cells. The percentage of cells in the subG1 peak observed in treated leukemia cells was higher respect to that observed in solid tumors cells, and the cleavage of PARP1 was evident after shorter time of exposure respect to melanoma and NSCLC cells, thus sustaining the evidence of a higher sensitivity of leukemia cells to IS20 and IS21 exposure, in agreement with the important role played by Bcl-2 in hematological malignancies 25.

There is a great interest in understanding the relationship between apoptosis and autophagy, particularly due the controversial role of autophagy in cancer. Autophagy has been reported to activate programmed cell death, as well as, to act as survival mechanism in several tumor types 41. It may represent a death program in resistant tumor cells 41, hence the identification of new drugs that specifically trigger autophagic cell death is a big challenge in cancer research. Moreover, the development of new autophagy-inducing agents may be useful also for preventing and/or treating other diseases in which autophagy is compromised. The interaction between Bcl-2 family proteins, including Bcl-2 and Beclin 1, the most studied player of autophagy, has been described among the mechanism involved in the crosstalk between autophagy and apoptosis 21, 43, 45, 47. In agreement, BDA-366 and the pan Bcl-2 family inhibitor AT-101 have been reported to significantly induce autophagic program in human lung cancer and osteosarcoma cells, respectively 21, 56. Interestingly, we showed that the autophagic flux was increased in melanoma and NSCLC cells upon IS20 and IS21 exposure, as evidenced by the ratio between LC3B-II on LC3B-I, as well as by autophagosome formation and maturation. Of note, the CQ chemical inhibitor of autophagy reverted IS21 effect on apoptosis and cell viability, thus indicating an autophagy-dependent mechanism of cell death and apoptosis induction. Interestingly, based on molecular docking results, we can sustain that IS21 undermines the binding to Beclin 1 similarly to other BH3-mimetic inhibitors, thus increasing autophagic program.

Molecular docking simulations showed IS21 to bind Bcl-2 protein resembling ABT-199 experimental binding mode. Indeed, IS21 bound conformation was not conformationally affected by G101V, G101A and F104L mutations, among which G101V was reported to be related to ABT-199 resistance, together with mutations involving other residues 27, 28. Binding mode inspection revealed IS21 mimicking the four residues of the Beclin 1 BH3 α-helix similarly as reported for ABT-199. Molecular docking simulation carried out also into Bcl-xL and Mcl-1 proteins revealed for IS21 lower docking energies for Bcl-2 and Bcl-xL than for Mcl-1. Nevertheless, IS21 proved to be, both experimentally (SPR assay) and virtually, a flexible molecule able to bind the three anti-apoptotic proteins. Moreover, the *in silico* ADMET profile of IS21 revealed some advantages in respect to that calculated on the reference compound ABT-199, indicating IS21 as a promising lead compound to be further studied and optimized. Finally, when intraperitoneally injected in mice carrying leukemia or melanoma tumors, IS21 significantly reduced tumor growth in both preclinical models, while it did not cause any adverse health effects. Furthermore, when IS21 was compared *in vivo* with ABT-199 and ABT-263, it showed a similar anti-tumoral effect against A375 model.

In summary, we have identified in the IS21 compound a pan ligand for Bcl-2, Bcl-xL and Mcl-1 anti-apoptotic members, which exhibits potent *in vitro* and *in vivo* efficacy against human leukemia and melanoma cancer models, offering the potential for further preclinical investigation. Drug design efforts are currently ongoing for a lead optimization campaign to select new molecules candidates endowed of either selective or pan affinity for the Bcl-2 family proteins.

## Supplementary Material

Supplementary methods, figures, and tables.

Additional files.

## Figures and Tables

**Table 1:** KD of the 8 most promising compounds for Bcl-2, Bcl-xL and Mcl-1 proteins. * Two compounds most investigated in the study. The chemical structures of IS20 and IS21 are also depicted.

COMPOUND	Bcl-2 (µM)	Bcl-xL (µM)	Mcl-1 (µM)
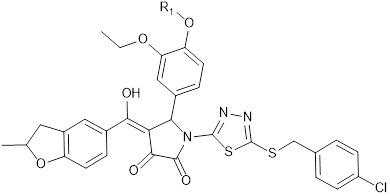 IS20: R1 = -CH_2_-CH_2_-CH_2_-CH_3_ IS21: R1 = -CH_2_-CH_2_-CH_2_-CH_2_-CH_3_			
IS21*	0.19 ± 0.07	0.51 ± 0.17	1.16 ± 0.14
IS20*	0.32 ± 0.09	0.42 ± 0.09	3.9 ± 0.30
IS1	0.48 ± 0.11		
ISQ	0.53 ± 0.10		
ISP	0.77 ± 0.14		
IS29	3.40 ± 1.10		
IS9	4.00 ± 0.70		
IS27	4.60 ± 0.90		

## Abbreviations

ADMET: Adsorption, Distribution, Metabolism, Excretion
AML: acute myeloid leukemia
APL: acute promyelocytic leukemia
ATP: Adenosine TriPhosphate
AUC: area under the curve
BA Class: biochemical based assay
Bcl-2: B-cell lymphoma-2
CLL: chronic lymphocytic leukemia
DMPK: Drug, Metabolism, Pharmacokinetics
DMSO: Dimethyl sulfoxide
FA Class: functional cell based assay
HSP72/73: heat shock-related protein
LC3-B: 1A/1B-light chain 3 protein
MCC: Matthew correlation coefficient
ML: machine learning
MTT: 3-[4,5-dimethylthiazol-2-yl]-2,5-diphenyltetrazolium bromide inner salt
NSCLC: non-small cell lung cancer
PARP: poly (ADP-ribose) polymerase
PEG 400: polyethylene glycol 400
PI: propidium iodide
ptf-LC3: pH-sensitive, double-tagged mRFP-GFP-LC3B reporter
QSAR: quantitative structure-activity relationship
RU: Response Units
SCLC: small cell lung cancer
SD: standard deviation
SEM: standard error of the mean
SLL: small lymphocytic lymphoma
SPR: Surface Plasmon Resonance
VS: virtual screening

## References

[1] Kale J, Osterlund EJ, Andrews DW (2018). BCL-2 family proteins: changing partners in the dance towards death. Cell Death Differ.

[2] Gross A, Katz SG (2017). Non-apoptotic functions of BCL-2 family proteins. Cell Death Differ.

[3] Adams JM, Cory S (2018). The BCL-2 arbiters of apoptosis and their growing role as cancer targets. Cell Death Differ.

[4] Trisciuoglio D, Iervolino A, Zupi G, Del Bufalo D (2005). Involvement of PI3K and MAPK signaling in bcl-2-induced vascular endothelial growth factor expression in melanoma cells. Mol Biol Cell.

[5] Trisciuoglio D, Desideri M, Ciuffreda L, Mottolese M, Ribatti D, Vacca A (2005). Bcl-2 overexpression in melanoma cells increases tumor progression-associated properties and *in vivo* tumor growth. J Cell Physiol.

[6] Trisciuoglio D, Gabellini C, Desideri M, Ziparo E, Zupi G, Del Bufalo D (2010). Bcl-2 regulates HIF-1alpha protein stabilization in hypoxic melanoma cells via the molecular chaperone HSP90. PLoS One.

[7] Trisciuoglio D, Gabellini C, Desideri M, Ragazzoni Y, De Luca T, Ziparo E (2011). Involvement of BH4 domain of bcl-2 in the regulation of HIF-1-mediated VEGF expression in hypoxic tumor cells. Cell Death Differ.

[8] Gabellini C, De Luca T, Trisciuoglio D, Desideri M, Di Martile M, Passeri D (2013). BH4 domain of bcl-2 protein is required for its proangiogenic function under hypoxic condition. Carcinogenesis.

[9] De Luca T, Pelosi A, Trisciuoglio D, D'Aguanno S, Desideri M, Farini V (2016). miR-211 and MITF modulation by Bcl-2 protein in melanoma cells. Mol Carcinog.

[10] Trisciuoglio D, Desideri M, Farini V, De Luca T, Di Martile M, Tupone MG (2016). Affinity purification-mass spectrometry analysis of bcl-2 interactome identified SLIRP as a novel interacting protein. Cell Death Dis.

[11] Trisciuoglio D, Tupone MG, Desideri M, Di Martile M, Gabellini C, Buglioni S (2017). BCL-XL overexpression promotes tumor progression-associated properties. Cell Death Dis.

[12] D'Aguanno S, Valentini E, Tupone MG, Desideri M, Di Martile M, Spagnuolo M (2018). Semaphorin 5A drives melanoma progression: role of Bcl-2, miR-204 and c-Myb. J Exp Clin Cancer Res.

[13] Tupone MG, D'Aguanno S, Di Martile M, Valentini E, Desideri M, Trisciuoglio D (2020). microRNA-378a-5p iS a novel positive regulator of melanoma progression. Oncogenesis.

[14] Di Martile M, Farini V, Consonni FM, Trisciuoglio D, Desideri M, Valentini E (2020). Melanoma-specific bcl-2 promotes a protumoral M2-like phenotype by tumor-associated macrophages. J Immunother Cancer.

[15] Giorgini S, Trisciuoglio D, Gabellini C, Desideri M, Castellini L, Colarossi C (2007). Modulation of bcl-xL in tumor cells regulates angiogenesis through CXCL8 expression. Mol Cancer Res.

[16] Gabellini C, Castellini L, Trisciuoglio D, Kracht M, Zupi G, Del Bufalo D (2008). Involvement of nuclear factor-kappa B in bcl-xL-induced interleukin 8 expression in glioblastoma. J Neurochem.

[17] Gabellini C, Gomez-Abenza E, Ibanez-Molero S, Tupone MG, Perez-Oliva AB, de Oliveira S (2018). Interleukin 8 mediates bcl-xL-induced enhancement of human melanoma cell dissemination and angiogenesis in a zebrafish xenograft model. Int J Cancer.

[18] Choi S, Chen Z, Tang LH, Fang Y, Shin SJ, Panarelli NC (2016). Bcl-xL promotes metastasis independent of its anti-apoptotic activity. Nat Commun.

[19] Bedikian AY, Garbe C, Conry R, Lebbe C, Grob JJ, Genasense Melanoma Study G (2014). Dacarbazine with or without oblimersen (a Bcl-2 antisense oligonucleotide) in chemotherapy-naive patients with advanced melanoma and low-normal serum lactate dehydrogenase: 'The AGENDA trial'. Melanoma Res.

[20] Gabellini C, Trisciuoglio D, Del Bufalo D (2017). Non-canonical roles of Bcl-2 and Bcl-xL proteins: relevance of BH4 domain. Carcinogenesis.

[21] Liu Z, Wild C, Ding Y, Ye N, Chen H, Wold EA (2016). BH4 domain of Bcl-2 as a novel target for cancer therapy. Drug Discov Today.

[22] Garner TP, Lopez A, Reyna DE, Spitz AZ, Gavathiotis E (2017). Progress in targeting the BCL-2 family of proteins. Curr Opin Chem Biol.

[23] Wilson WH, O'Connor OA, Czuczman MS, LaCasce AS, Gerecitano JF, Leonard JP (2010). Navitoclax, a targeted high-affinity inhibitor of BCL-2, in lymphoid malignancies: a phase 1 dose-escalation study of safety, pharmacokinetics, pharmacodynamics, and antitumour activity. Lancet Oncol.

[24] Zhang H, Nimmer PM, Tahir SK, Chen J, Fryer RM, Hahn KR (2007). Bcl-2 family proteins are essential for platelet survival. Cell Death Differ.

[25] Souers AJ, Leverson JD, Boghaert ER, Ackler SL, Catron ND, Chen J (2013). ABT-199, a potent and selective BCL-2 inhibitor, achieves antitumor activity while sparing platelets. Nat Med.

[26] Juarez-Salcedo LM, Desai V, Dalia S (2019). Venetoclax: evidence to date and clinical potential. Drugs Context.

[27] Birkinshaw RW, Gong JN, Luo CS, Lio D, White CA, Anderson MA (2019). Structures of BCL-2 in complex with venetoclax reveal the molecular basis of resistance mutations. Nat Commun.

[28] Salah HT, DiNardo CD, Konopleva M, Khoury JD (2021). Potential Biomarkers for Treatment Response to the BCL-2 Inhibitor Venetoclax: State of the Art and Future Directions. Cancers (Basel).

[29] Lochmann TL, Bouck YM, Faber AC (2018). BCL-2 inhibition is a promising therapeutic strategy for small cell lung cancer. Oncoscience.

[30] Ragno R, Gioia U, Laneve P, Bozzoni I, Mai A, Caffarelli E (2011). Identification of small-molecule inhibitors of the XendoU endoribonucleases family. ChemMedChem.

[31] Laneve P, Gioia U, Ragno R, Altieri F, Di Franco C, Santini T (2008). The tumor marker human placental protein 11 is an endoribonuclease. J Biol Chem.

[32] Ballante F, Caroli A, Wickersham RB 3rd, Ragno R (2014). Hsp90 inhibitors, part 1: definition of 3-D QSAutogrid/R models as a tool for virtual screening. J Chem Inf Model.

[33] Caroli A, Ballante F, Wickersham RB 3rd, Corelli F, Ragno R (2014). Hsp90 inhibitors, part 2: combining ligand-based and structure-based approaches for virtual screening application. J Chem Inf Model.

[34] Neves BJ, Braga RC, Melo-Filho CC, Moreira-Filho JT, Muratov EN, Andrade CH (2018). QSAR-Based Virtual Screening: Advances and Applications in Drug Discovery. Front Pharmacol.

[35] Gaulton A, Bellis LJ, Bento AP, Chambers J, Davies M, Hersey A (2012). ChEMBL: a large-scale bioactivity database for drug discovery. Nucleic Acids Res.

[36] Kim S, Thiessen PA, Cheng T, Zhang J, Gindulyte A, Bolton EE (2019). PUG-View: programmatic access to chemical annotations integrated in PubChem. J Cheminform.

[37] Trisciuoglio D, De Luca T, Desideri M, Passeri D, Gabellini C, Scarpino S (2013). Removal of the BH4 domain from Bcl-2 protein triggers an autophagic process that impairs tumor growth. Neoplasia.

[38] Di Martile M, Desideri M, De Luca T, Gabellini C, Buglioni S, Eramo A (2016). Histone acetyltransferase inhibitor CPTH6 preferentially targets lung cancer stem-like cells. Oncotarget; Vol 7, No 10.

[39] Genovese I, Fiorillo A, Ilari A, Masciarelli S, Fazi F, Colotti G (2017). Binding of doxorubicin to Sorcin impairs cell death and increases drug resistance in cancer cells. Cell Death & Disease.

[40] Genovese I, Giamogante F, Barazzuol L, Battista T, Fiorillo A, Vicario M (2020). Sorcin is an early marker of neurodegeneration, Ca(2+) dysregulation and endoplasmic reticulum stress associated to neurodegenerative diseases. Cell Death Dis.

[41] Mulcahy Levy JM, Thorburn A (2020). Autophagy in cancer: moving from understanding mechanism to improving therapy responses in patients. Cell Death Differ.

[42] Lee EF, Smith NA, Soares da Costa TP, Meftahi N, Yao S, Harris TJ (2019). Structural insights into BCL2 pro-survival protein interactions with the key autophagy regulator BECN1 following phosphorylation by STK4/MST1. Autophagy.

[43] Nhu D, Lessene G, Huang DCS, Burns CJ (2016). Small molecules targeting Mcl-1: the search for a silver bullet in cancer therapy. MedChemComm.

[44] Liu X, Zhang Y, Huang W, Tan W, Zhang A (2018). Design, synthesis and pharmacological evaluation of new acyl sulfonamides as potent and selective Bcl-2 inhibitors. Bioorganic & Medicinal Chemistry.

[45] Merino D, Kelly GL, Lessene G, Wei AH, Roberts AW, Strasser A (2018). BH3-Mimetic Drugs: Blazing the Trail for New Cancer Medicines. Cancer Cell.

[46] Inoue-Yamauchi A, Jeng PS, Kim K, Chen HC, Han S, Ganesan YT (2017). Targeting the differential addiction to anti-apoptotic BCL-2 family for cancer therapy. Nat Commun.

[47] Han B, Park D, Li R, Xie M, Owonikoko TK, Zhang G (2015). Small-Molecule Bcl2 BH4 Antagonist for Lung Cancer Therapy. Cancer Cell.

[48] Birkinshaw RW (2021). Challenges in small-molecule target identification: a commentary on "BDA-366, a putative Bcl-2 BH4 domain antagonist, induces apoptosis independently of Bcl-2 in a variety of cancer cell models". Cell Death Differ.

[49] Karpel-Massler G, Ishida CT, Bianchetti E, Shu C, Perez-Lorenzo R, Horst B (2017). Inhibition of Mitochondrial Matrix Chaperones and Antiapoptotic Bcl-2 Family Proteins Empower Antitumor Therapeutic Responses. Cancer Res.

[50] Serasinghe MN, Gelles JD, Li K, Zhao L, Abbate F, Syku M (2018). Dual suppression of inner and outer mitochondrial membrane functions augments apoptotic responses to oncogenic MAPK inhibition. Cell Death Dis.

[51] Xu W, Guo G, Li J, Ding Z, Sheng J, Li J (2016). Activation of Bcl-2-Caspase-9 Apoptosis Pathway in the Testis of Asthmatic Mice. PLOS ONE.

[52] Schulze BA, Evers G, Kerkhoff A, Mohr M, Schliemann C, Berdel EW (2019). Future Options of Molecular-Targeted Therapy in Small Cell Lung Cancer. Cancers.

[53] Mathews Griner LA, Zhang X, Guha R, McKnight C, Goldlust IS, Lal-Nag M (2016). Large-scale pharmacological profiling of 3D tumor models of cancer cells. Cell Death Dis.

[54] Abdullah LN, Chow EK (2013). Mechanisms of chemoresistance in cancer stem cells. Clin Transl Med.

[55] Sakariassen PO, Immervoll H, Chekenya M (2007). Cancer stem cells as mediators of treatment resistance in brain tumors: status and controversies. Neoplasia.

[56] Masuelli L, Benvenuto M, Izzi V, Zago E, Mattera R, Cerbelli B (2019). *In vivo* and *in vitro* inhibition of osteosarcoma growth by the pan Bcl-2 inhibitor AT-101. Invest New Drugs.

