# Supplementary material for: Targeting the anti-apoptotic Bcl-2 family proteins: machine learning virtual screening and biological evaluation of new small molecules
Source: Theranostics. 2022 Feb 28;12(5):2427–44. doi: 10.7150/thno.64233 (PMC8899577; doi:10.7150/thno.64233)
Supplement: Supplementary file 1 — Supplementary methods, figures, and tables. [file thnov12p2427s1.pdf]

Supplementary Information

Targeting the anti-apoptotic Bcl-2 family proteins: machine learning virtual screening and biological evaluation of new small molecules

Elisabetta Valentini[1,*], Simona D'Aguanno[1,*], Marta Di Martile[1,*], Camilla Montesano[2], Virginia Ferraresi[3], Alexandros Patsilinakos[4,5], Manuela Sabatino[4,5], Lorenzo Antonini[4,5], Martina Chiacchiarini[1], Sergio Valente[5], Antonello Mai[5,6], Gianni Colotti[7], Rino Ragno[4,5]✉, Daniela Trisciuoglio[1,7,]✉, Donatella Del Bufalo[1,]✉

[1]Preclinical Models and New Therapeutic Agents Unit, IRCCS Regina Elena National Cancer Institute, Via Elio Chianesi 53, Rome, Italy;
[2]Department of Chemistry, Sapienza University of Rome, P.le Aldo Moro 5, Rome, Italy;
[3]Sarcomas and Rare Tumours Departmental Unit- IRCCS Regina Elena National Cancer Institute, Via Elio Chianesi 53, Rome, Italy;
[4]Rome Center for Molecular Design, Department of Drug Chemistry and Technologies, Sapienza University of Rome, P.le Aldo Moro 5, Rome, Italy;
[5]Department of Drug Chemistry and Technologies, Sapienza University of Rome, P.le Aldo Moro 5, Rome, Italy;
[6]Pasteur Institute, Cenci Bolognetti Foundation, Sapienza University of Rome, P.le Aldo Moro 5, Rome, Italy;
[7]Institute of Molecular Biology and Pathology, Italian National Research Council, P.le A.Moro 5, Rome, Italy.

[*] Equal contribution as first authors: Elisabetta Valentini, Simona D'Aguanno, Marta Di Martile
✉ Corresponding authors: Donatella Del Bufalo, Daniela Trisciuoglio, Rino Ragno

SUPPLEMENTARY METHODS

<u>Virtual screening protocol</u>

A ligand-based (LB) VS protocol was set up to select potential Bcl-2 modulators compounds from a ~ 1 million of compounds commercial database. The study started with the compilation of a Bcl-2 modulators dataset interrogating the publicly available ChEMBL and PubChem databases and retrieving all records related to ligands assayed against Bcl-2 protein by either biochemical or cell-

based methods. The Bcl-2 modulators dataset was subjected to a pruning procedure and the cleaned dataset was opportunely divided into different training sets for the developing of predictive QSAR models to be used as filtering tools to rank a commercial small molecules database and acquire the most promising compounds to be experimentally tested as potential Bcl-2 modulators.

Data collection and pruning. A Bcl-2 modulators database was compiled from ChEMBL version 22 database. Human apoptosis regulator Bcl-2 bioactivity records, referred with the target identifier CHEMBL4860, were retrieved and processed to compile a dataset ($BCL2M_{ChEMBL}$ dataset). The initial $BCL2M_{ChEMBL}$ dataset of 1762 activity records was then subjected to a cleaning procedure (see material and methods section). A cleaned $BCL2M_{ChEMBL}$ dataset of 1634 compounds was obtained and divided into subsets: the binding assay set (BA) containing 1570 molecules and the functional assay one (FA) of 64 compounds. The two datasets (BA-$BCL2M_{ChEMBL}$ and FA-$BCL2M_{ChEMBL}$) were then used to build binary classification models.

Binary classification BA set (BA_{Class}) and FA Set (FA_{Class}). The 1570 BA-$BCL2M_{ChEMBL}$ compounds were divided into actives and non-actives (inactives) on the basis of a biological activity cutoff value of 1 μM (pAct = 6). Consequently 1167 were labeled actives as displayed K_i, K_d, IC_{50}, or EC_{50} values lower than 1 μM; whereas non-actives (K_i, K_d and IC_{50}, or $EC_{50} \geq 1$ μM) resulted to be 403. To balance actives and inactives ratio (actives/inactives = 2.90), further 923 molecules tagged as BCL2M were retrieved from PubChem (PC dataset) repository (geneID: 596). Cleaning of the PC dataset as above described led to a $BCL2M_{PC}$ dataset of 602 unique compounds that were merged with the $BCL2M_{ChEMBL}$ dataset leading to a more balanced actives/inactives ratio of 1.19 with 1180 active and 992 inactive compounds, respectively. A total number of 2172 datapoints was compiled for the BA_{Class}. In a similar fashion way the FA-$BCL2M_{ChEMBL}$ were classified into actives and inactives leading to a fully balanced training set composed of 32 active and 32 inactive molecules.

QSAR models building. QSAR were developed with ML techniques considering either classification methods. Molecular descriptor and fingerprints (DESCs and FPs) as calculated by means of RDKit (see experimental) were used as independent data (X_{DESCs} and X_{FPs}) in the following classification models' derivation, furthermore a combination of DESCs and FPs as a unique hybrid molecular descriptor (Hybrid) matrix were also used.

Evaluation of classification models. Several ML classification models were built with K-nearest neighbors (KNN) and logistic regression (LR), gradient bosting (GB), support vector machine (SVM) and random forest (RF) using as dependent variable vector the above defined datasets BA_{Class} and FA_{Class} datasets. Considering seven ML algorithm for each dataset a total of forty-two

classification models were derived as datasets' compounds were represented with FPs, DESCs and Hybrid (**Table S1-S6**).

As many of the under building models using default Hyperparameters settings displayed good values of accuracy (ACC) and Matthews correlation coefficient (MCC), to save computation time no hyperparameters' optimization was applied. As alternative to the model's optimization six further final consensus models were built, by means of the voting classifier utility of scikit-learn (sklearn) library [1] with the soft voting switch [2] (**Table S7).**

All consensus models showed a good propensity to classify actives from inactives, in particular FPs derived models displayed higher ACC and MCC values than those obtained with DESCs. In particular, the models obtained with the Hybrid combination displayed the highest internal stability and predictive power.

Molecular docking assessment. Any molecular docking program should to be evaluated for its ability in reproducing experimental co-crystallized complexes (when available). In this case as venetoclax was used as reference compound four complexes of it with *wild type* and mutate form of Bcl-2 were used (pdb entry codes: 6O0K, 6O0L, 6O0M_2 and 6O0P). To select the best performing docking program (DP) smina [3] and Plants [4] were selected as free for academics and among the most used. Based on the available feature (F) and scoring function (FS) nine different DP/F/FS combination were applied. On the basis of a random self-docking procedure [5] the smina program with the VINA scoring function as proved to be the most effective in reproducing the venetoclax experimental co-crystallized poses in four different complexes (**Table S11).**

As the lead compound IS21 was found to display to be active against the three Bcl-2 family proteins (Bcl-2, Bcl-xL and Mcl-1) a further assessment was performed for the Smina program by using complexes with a ligand able to bind all the three proteins. A survey on ChEMBL revealed navitoclax as tested for either Bcl-2 [6], Bcl-xL [6] or Mcl-1 [7], and being navitoclax structurally related to venetoclax the co-cristallized complexes with Bcl-2 (pdb entry code 6QGH) and Bcl-xL (pdb entry code 4QNQ) were retrieved from PDB. Docking assessment by means of re-docking and cross-docking experiments was run on the 6QGH and 4QNQ confirming the Smina/VINA combination the most suitable one (**Tables S12** and **S13**).

<u>Computational procedures</u>

Data collection and pruning. All BCL2M records were retrieved from ChEMBL and PubChem. Human apoptosis regulator Bcl-2 bioactivity records, referred with the target identifier. The initial $BCL2M_{ChEMBL}$ dataset was then subjected to a pruning procedure as following:

- compounds whose biological activity value was not expressed in IC_{50}, K_i, EC_{50}, GI_{50}, K_d, or biological data outside typical range were deleted;

- duplicates were aggregated by including only average pAct values showing standard deviations lower than 0.5;
- mixtures, inorganics, and organometallics were removed;
- all biological activities were converted into molar unit and transformed in logarithm scale by the p function (pAct = log([M]$^{-1}$);
- elimination of records flagged with alerts as "outside typical range" and "nonstandard unit of type" in the "data_validity_comment" field

The cleaned BCL2M$_{ChEMBL}$ dataset was divided into subsets: the binding assay set (BA) and the functional assay one (FA). The two sets (BA-BCL2M and FA-BCL2M) were then used to build binary classification and regression models.

Binary classification BA set (BA$_{Class}$) and ***FA Set (FA$_{Class}$)***. The BA-BCL2M compounds were divided into actives and non-actives (inactives) on the basis of an arbitrary pAct values of 6 (1 μM). In a similar fashion way the FA-BCL2M were classified into actives and inactives.

Molecule numerical representation. To build the QSAR models molecules were described in chemical and molecular representation using, molecular descriptors (DESCs, chemical description) and molecular fingerprints (Fps, structural description), respectively. DESCs and Fps were calculated by means of the RDKit python library (v. 2017.09.1). In particular Morgan type fingerprints were used by setting to 2048 the number of bits while using radius 3. Whereas all 200 RDKit descriptors were calculated for each molecule and scaled by means of the min-max method. DESCs and Fps were finally organized in matrixes to be used as independent data (X_{DESCs} and X_{FPs}) in the classification and regression models' derivation.

QSARs' building. All the steps related to the development of QSAR classification models and their VS application implemented in the Python version 3.5 programming environment using anaconda. The related code was written and executed in the jupyter-notebook [1] platform by including several libraries. Among the latter can be listed: numpy [2] and scipy [3] for numerical computing, pandas [4, 5] for data wrangling, scikit-learn for ML erlaboration, matplotlib [6] and plotly [7] for graphical output, RDKit and openbabel [8] for cheminformatics handlings.

For each QSAR classification models, was used as independent data the matrix above defined (X_{DESCs} or X_{MFPs}) with nohyperparameter optimization.

Classification modeling. Several binary classification models [9] were built with the following ML algorithms K-nearest neighbors (KNN) and logistic regression (LR), using as dependent variable vector the above defined datasets BA$_{Class}$ and FA$_{Class}$ datasets.

Models' performance internal evaluation. To evaluate the QSAR models generalization ability in predicting biological profile of new chemical entities, K-Fold cross validations were run for BA and

FA datasets. In particular, the leave half out method (LHO) was used and repeated for 100 iterations.

Finally, 1000 rounds of Y-scrambling were performed to assess any lack of chance correlation between.

Virtual screening. A database of about 1 million of compound was retrieved from a commercial vendor (VITAS-M) and subjected to the best QSAR models to rank the molecules and select the most promising as potential BCL2M (**Table S11**).

Molecular docking. All docking simulation were carried out with two free for academia programs, Smina and Plants considering all the possible combination of scoring function and minimization features (**Table S15**).

Selection of experimental Bcl-2 complexed with venetoclax for docking assessment. From a survey in the PDB database a list of four complexes (*wild* type and three mutated Bcl-2 proteins) of Bcl-2/venetoclax were found available (PDB entry codes: 6O0K, 6O0L, 6O0M_2 and 6O0P, **Table S16**)

The Structures were loaded in UCSF Chimera and aligned by means of the matchmaker module (mmaker).

Preparation of the selected proteins for the docking assessment. The selected complexes subjected to a cleaning protocol similarly as described (8-11). The cleaned complexes were then added of hydrogen with the addh module and geometrically optimized with a short single point minimization with the embedded AMBER minimization core and the ff14SB force field with 1000 steepest descent steps and 100 of conjugate gradient steps. The parameters of the complexed ligands were calculated applying the embedded antechamber module using the generalized force field (GAFF), using Gasteiger charges. At the end of the minimization all the complexes were realigned and stored in PDB and MOL2 formats files. Each of all the minimized and aligned complexes were then separed into protein and ligand (venetoclax). PDBQT file were generated from MOL2 by means of command line tools available fromAutoDock Tools 1.56 (ADT).

Molecular docking settings. Either Smina or Plants require to define the space to run the docking. To this, as all the complexes contained a well superimposed venetoclax conformation, the coordinate of just on venetoclax was used to define the docking space. To run the docking configuration files were prepared for either Smina (**Table S17**) and Plants (**Table S18**). For Plants the binding site center (bindingsite_center 9.0 3.0 -7.0) it was defined by the reference.pdb center of mass, while the binding site radius (bindingsite_radius 21.0) was calculated adding 5Å to the half of the longer dimension of the least box containing the reference.pdb file itself.

Docking affinities as calculated by the programs were stored and root mean square deviation (RMSD) were evaluated by means of the pkcombu program and used to calculate the docking accuracies.

IS20 and IS21 were docked into 6QGH (Bcl-2) and 4QNQ (Bcl-xL) proteins, and due to the lack of any complex of Mcl-1 with navitoclax the pdb entry code 6YBL was retrieved as co-cristallized with the a ligand structure closest to that of venetoclax. The latter was used to predict the IS20 and IS21 binding conformations into Mcl-1. Due to the high flexibility of the Bcl-2 family proteins three further complexes of Bcl-2, Bcl-xL and Mcl-1 complexed with BH3 were also retrieved (4B4S, 4QVF and 6QFI, respectively) to explore for alternative binding modes (**Tables S12** and **S13**).

Structure preparation of interesting active compounds. The structure of IS21, IS20, IS1, ISQ, ISP, IS9, IS27 and IS36 were available in SMILES format and then were converted into PDB and MOL2 (for docking with Plants) formats files with openbabel 2.41 using the –gen3D option. Finally, the PDBQT (for docking with smina) formats were obtained by means of the ADT command line tools.

<u>Pharmacokinetic</u>

***In vivo* experiments.** To evaluate the pharmacokinetic profile of IS20 and IS21, C57/Bl6 mice were intraperitoneally injected with a single dose of IS20 or IS21 (100 mg/kg) dissolved in 10% DMSO, 30% PEG400 (Sigma-Aldrich) and 60% NaCl. Mice were sacrificed starting from 15 min to 24 h after injection. Blood samples were collected by cardiac puncture, centrifuged at 3,000 g for 10 min at 4 °C and the obtained plasma was stored at -80 °C until the analysis performed by liquid chromatography tandem mass spectrometry (LC–MS/MS). Given that the two compounds were singularly administered to the animals for the pharmacokinetic studies, IS20 was used as internal standard (IS) for IS21 and vice versa. Internal Standard Working Solutions (IS-WS) were prepared by adding appropriate volumes of the stock solutions to 50 mL of 0.1% formic acid (Fluka, Milan, Italy) in methanol:acetonitrile (50:50, v/v, Sigma-Aldrich), in order to reach a final concentration of 50 ng/mL. The solutions were maintained at -20 °C.

LC-MS/MS Analysis. 40 µL of mouse plasma were mixed with 160 µL of IS-WS. The mixture was vortex mixed for 1 min and centrifuged at 12,000 g for 10 min at 4 °C. The supernatant was collected and 8 µL were injected into the LC–MS/MS system. The HPLC equipment consists of a Series 200 Micro-LC Pump and a Series 200 autosampler from Perkin Elmer (Norwalk, CT, USA). A triple quadrupole mass spectrometer, AB-Sciex API2000 (Toronto, ON, Canada) was used for detection. The analytes were analyzed using a C_{18} phase Kinetex column (10 cm x 2.1 mm ID) from Phenomenex (Torrance, CA, USA) packed with core–shell particles of 2.6 µM. The mobile phases were (A) acetonitrile and (B) water, both containing 0.1% formic acid, at a flow rate of 0.25 mL/

min and were entirely transferred into the mass spectrometer source. Gradient elution was as follows: increase of the organic phase from 60 to 80% in 0.8 min, then to 90% in the following 1.2 min and linearly to 100% in 2 min. Finally, after 3.9 min of 100%, the column was led to the original conditions in 2.5 min to enable equilibration of the column.

Both analytes were detected in positive ionization with a capillary voltage of 5500 V, nebulizer gas (air) at 40 psi, turbo gas (nitrogen) at 70 psi and 400 °C. The other ion source parameters were set as follows: curtain gas (CUR) 18 psi; collision gas (CAD) 6 psi; declustering potential (DP) 80 V, entrance potential (EP) 12 V.

The quantitative data were acquired using Multi Reaction Monitoring (MRM) acquisition mode. Two MRM transitions (precursor ion>fragment ion) were selected for the analytes. For IS20 transitions were *m/z* 692.3 > 407.3 and 692.3 > 161.1, collision energy (CE) was set at 30 and 52 eV while collision cell exit potential (CXP) was at 19 and 8 V for the two transitions, respectively. For IS21 transitions were *m/z* 706.3 > 421.3 and 706.3 > 160.7, collision energy (CE) was set at 30 and 51 eV while collision cell exit potential (CXP) was at 21 and 6 V for the two transitions, respectively.

The analytical method was validated according to FDA guidelines for bioanalytical method validation. Linearity, recovery, matrix effect, precision, accuracy, limits of detection (LODs) and lower limits of quantification (LLOQs) were evaluated.

Calibration standard solutions were prepared in blank plasma by spiking 25 µL of a standard mixture at appropriate concentration to 40 µL of plasma and by adding 140 µL of methanol: acetonitrile (50:50, v/v). Calibrators were then treated similarly to the animal samples. The calibration range was 5 to 7500 ng/mL and the calibrators were prepared at nine level of concentration. Precision, recovery and accuracy were evaluated at three level of concentrations (25, 25, 5000 ng/mL) and resulted within the acceptable limits.

The limit of detection (LOD) was defined as the lowest concentration with a signal-to-noise (S/N) ratio greater than 3. The limit of quantification (LOQ) was defined as the concentration at which both precision (RSD%) and accuracy were less than 20%. LOQ resulted to be 2 ng/mL while LOD was 0.5 ng/mL.

The validated method was then successfully applied in measuring IS20 and IS21 following drug administration in mice plasma to support the pharmacokinetic study (**Figure S8A,B**).

SUPPLEMENTARY TABLES

Table S1. *Accuracy (ACC) and Matthews correlation coefficient (MCC) for the ML models developed with* FA_{Class} *dataset and DESCs.*

ML method	Fitting		Cross-Validation	
	**ACC**	**MCC**	**ACC**	**MCC**
RandomForest	0.99	0.97	0.83	0.62
GradientBoosting	0.99	0.97	0.87	0.71
SVM_linearL1	0.79	0.55	0.73	0.41
SVM_linearL2	0.79	0.54	0.77	0.51
LogisticRegressionL1	0.87	0.71	0.81	0.59
LogisticRegressionL2	0.91	0.80	0.79	0.53
KNeighbors	0.88	0.74	0.81	0.60

Table S2. *Accuracy (ACC) and Matthews correlation coefficient (MCC) for the ML models developed with* FA_{Class} *dataset and FPs.*

ML method	Fitting		Cross-Validation	
	**ACC**	**MCC**	**ACC**	**MCC**
RandomForest	1.00	1.00	0.83	0.62
GradientBoosting	1.00	1.00	0.83	0.62
SVM_linearL1	0.88	0.74	0.79	0.53
SVM_linearL2	0.95	0.89	0.83	0.62
LogisticRegressionL1	0.96	0.92	0.83	0.62
LogisticRegressionL2	1.00	1.00	0.83	0.62
KNeighbors	0.91	0.80	0.84	0.65

Table S3. *Accuracy (ACC) and Matthews correlation coefficient (MCC) for the ML models developed with* FA_{Class} *dataset and Hybrid (see text).*

ML method	Fitting		Cross-Validation	
	**ACC**	**MCC**	**ACC**	**MCC**
RandomForest	1.00	1.00	0.85	0.68
GradientBoosting	1.00	1.00	0.85	0.68
SVM_linearL1	0.84	0.67	0.77	0.50
SVM_linearL2	0.97	0.94	0.81	0.59
LogisticRegressionL1	0.96	0.92	0.80	0.56
LogisticRegressionL2	1.00	1.00	0.83	0.62
KNeighbors	0.91	0.80	0.80	0.56

Table S4. *Accuracy (ACC) and Matthews correlation coefficient (MCC) for the ML models developed with* BA_{Class} *dataset and DESCs.*

ML method	Fitting		Cross-Validation	
	**ACC**	**MCC**	**ACC**	**MCC**

RandomForest	0.96	0.92	1.00	1.00
GradientBoosting	0.96	0.91	0.98	0.95
SVM_linearL1	0.95	0.90	0.96	0.92
SVM_linearL2	0.95	0.89	0.96	0.91
LogisticRegressionL1	0.95	0.89	0.96	0.91
LogisticRegressionL2	0.95	0.89	0.96	0.91
KNeighbors	0.95	0.91	0.97	0.93

Table S5. *Accuracy (ACC) and Matthews correlation coefficient (MCC) for the ML models developed with BA_{Class} dataset and FPs.*

ML method	Fitting		Cross-Validation	
	ACC	MCC	ACC	MCC
RandomForest	1.00	1.00	0.96	0.91
GradientBoosting	0.97	0.93	0.95	0.90
SVM_linearL1	0.98	0.96	0.95	0.91
SVM_linearL2	0.98	0.96	0.95	0.91
LogisticRegressionL1	0.99	0.98	0.95	0.91
LogisticRegressionL2	1.00	1.00	0.96	0.92
KNeighbors	0.97	0.93	0.95	0.89

Table S6. *Accuracy (ACC) and Matthews correlation coefficient (MCC) for the ML models developed with BA_{Class} dataset and FPs.*

ML method	Fitting		Cross-Validation	
	ACC	MCC	ACC	MCC
RandomForest	1.00	1.00	0.96	0.92
GradientBoosting	0.97	0.95	0.96	0.91
SVM_linearL1	0.98	0.96	0.95	0.90
SVM_linearL2	0.99	0.97	0.95	0.91
LogisticRegressionL1	0.99	0.98	0.96	0.91
LogisticRegressionL2	1.00	1.00	0.96	0.91
KNeighbors	0.97	0.93	0.95	0.89

Table S7. *Accuracy (ACC) and Matthews correlation coefficient (MCC) for the ML models developed with the soft voting classifier.*

Model combination	Fitting		Cross-Validation	
	ACC	MCC	ACC	MCC
FA_{Class} dataset and DESCs	0.97	0.94	0.83	0.62
FA_{Class} dataset and FPs	1.00	1.00	0.81	0.60
FA_{Class} dataset and Hybrid	1.00	1.00	0.84	0.65
BA_{Class} dataset and DESCs	0.97	0.95	0.96	0.89
BA_{Class} dataset and FPs	0.99	0.96	0.96	0.90
BA_{Class} dataset and Hybrid	0.99	0.98	0.96	0.91

Table S8. *List of 49 screened compounds. In bold are reported the 8 most effective compounds. *Two most characterized compounds (IS20 and IS21).*

ARBITRARY CODE	VITAS-M ID	ARBITRARY CODE	VITAS-M ID
**IS1**	**STK066520**	**IS29**	**STL341576**
IS3	STK893094	IS30	STK541283
IS5	STK145249	IS31	STK549022
IS6	STK398814	IS32	STL049462
IS7	STL168349	IS33	STL052557
IS8	STL168606	IS34	STK550725
**IS9**	**STL481050**	IS35	STK145055
IS10	STL481045	IS37	STL429533
IS11	STK060635	ISA	STK366292
IS13	STK595018	ISB	STK367596
IS14	STK548699	ISC	STK394848
IS15	STK673714	ISD	STK362856
IS16	STL057856	ISE	STK146847
IS17	STL052719	ISF	STL132867
IS18	STK551574	ISG	STK194419
IS19	STK554551	ISH	STK192532
**IS20 ***	**STK569102**	ISI	STK389854
**IS21 ***	**STK570207**	ISL	STK332010
IS23	STK584185	ISO	STL380528
IS24	STK593354	**ISP**	**STL181070**
IS25	STK597501	**ISQ**	**STL173124**
IS26	STK792977	ISR	STK117446
**IS27**	**STL333693**	ISS	STK115790
IS28	STL337517	IST	STK237001
		ISU	STK237006

Table S9. *Predicted docked energy by the VINA scoring function for IS21 as docked into Bcl-2, Bcl-xL and Mcl-1 proteins extracted from complexes containing small molecule ligands (pdb entry codes: 6QGH, 4QNQ and 6YBL) and from complexes containing the BIM BH3 a-helix (pdb entry codes: 4BAS, 4QVF and 6QFI).*

Bcl Protein Type	pdb entry code	Docking Energy (kcal/mol)		KD_1 (μM)	
		IS21	IS20	IS21	IS20
Bcl-2	6QGH	-8.64	-8.29	0.32	0.19
	4B4S	-7.86	-7.40		
	Average	-8.25	-7.85		
Bcl-xL	4QNQ	-8.67	-8.25	0.42	0.51
	4QVF	-8.26	-8.16		
	Average	-8.46	-8.21		
Mcl-1	6YBL	-7.65	-7.53	3.90	1.16
	6QFI	-8.09	-6.93		
	Average	-7.87	-7.23		

Table S10. *ADMET parameter as calculated by mean of the swissadme web too l* [13].

Molecule	IS20	IS21	venetoclax
MW	692.24	706.27	868.44
#Heavy atoms	47	48	61
#Aromatic heavy atoms	23	23	27
Fraction Csp3	0.31	0.33	0.38
#Rotatable bonds	12	13	14
#H-bond acceptors	8	8	9
#H-bond donors	1	1	3
MR	188.2	193.01	246.7
TPSA	164.62	164.62	183.09
MLOGP	3.1	3.69	3.22
ESOL Log S	-8.9	-9.26	-9.78
ESOL Solubility (mg/ml)	0.000000872	0.000000389	0.000000144
ESOL Solubility (mol/l)	1.26E-09	5.5E-10	1.65E-10
ESOL Class	Poorly soluble	Poorly soluble	Poorly soluble
Silicos-IT Solubility (mol/l)	1.14E-11	4.72E-12	4.45E-14
Silicos-IT class	Insoluble	Insoluble	Insoluble
GI absorption	Low	Low	Low
BBB permeant	No	No	No
Pgp substrate	Yes	Yes	Yes
CYP1A2 inhibitor	No	No	No
CYP2C19 inhibitor	Yes	Yes	No
CYP2C9 inhibitor	No	No	No
CYP2D6 inhibitor	No	Yes	No
CYP3A4 inhibitor	No	No	No
log Kp (cm/s)	-4.67	-4.36	-5.79
Lipinski #violations	1	1	2
Synthetic Accessibility	5.77	5.89	6.05

Table S11. *Root mean squared deviations (RMSD) in the re-docking assessment of venetoclax in four experimental complexes (wild* type and three mutated Bcl-2 proteins). *The mean values are also reported.*

**PDB ID**	**Smina**						**Plants**		
	**VINA**		**VINARDO**		**AD4_SCORING**				
	**BD***	**BD_{Min}****	**BD**	**BD_{Min}**	**BD**	**BD_{Min}**	**PLP**	**PLP95**	**CHEMPLP**
6O0K	1.33	1.31	1.32	1.32	1.32	1.31	1.69	1.59	6.78
6O0L	1.61	1.62	1.65	1.63	1.65	1.66	1.59	1.79	1.70
6O0M	1.15	1.16	1.20	1.24	1.16	1.74	2.34	2.08	2.16
6O0P	1.25	1.25	1.24	1.25	1.26	1.10	0.63	1.25	5.57
Mean	1.34	1.34	1.35	1.36	1.35	1.45	1.56	1.68	4.05

*BD: Best Docked conformation; **BD_{Min}: minimized BD; VINA, VINARDO, AD4_SCORING are the scoring function available in smina; PLP, PLP95 and CHEMPLP are the scoring function available in Plants.

Table S12. *Re-docking assessment for the Smina molecular docking program for the navitoclax Bcl-2 and Bcl-xL complexes. Root mean squared deviations (RMSD) and their mean values are also reported.*

**scoring**	**pdb entry code**		**ECRD**	**ECRD Min**	**RCRD**	**RCRD Min**
	**Ligand**	**Protein**				
AD4_SCORING	6QGH	6QGH	2.21	1.39	3.09	3.38
	4QNQ	4QNQ	5.04	5.19	3.37	3.46
	Average		3.62	3.29	3.23	3.42
	Docking Accuracy		25	50	25	25
VINA	6QGH	6QGH	2.31	1.34	2.55	4.00
	4QNQ	4QNQ	1.94	1.59	1.35	3.95
	Average		2.12	1.46	1.95	3.97
	Docking Accuracy		75	100	75	25
VINARDO	6QGH	6QGH	2.24	1.15	5.36	5.68
	4QNQ	4QNQ	1.41	1.41	1.16	5.18
	Average		1.82	1.28	3.26	5.43
	Docking Accuracy		75	100	50	0

ECRD: experimental conformation re-docking; ECRD: experimental conformation re-docking after minimization; RCRD: random conformation re-docking; docking; RCRD: random conformation re-dockin after minimization; VINA, VINARDO, AD4_SCORING are the scoring function available in smina; pdb entry code: PDB codes for Bcl-2 and Bcl-xL proteins co-crystallized with navitoclax

Table S13. *Cross-docking assessment for the Smina molecular docking program for the navitoclax Bcl-2 and Bcl-xL complexes. Root mean squared deviations (RMSD) and their mean values are also reported*

scoring	pdb entry code		ECRD	ECRD Min	RCRD	RCRD Min
	Ligand	Protein				
AD4_SCORING	6QGH	4QNQ	4.11	4.54	3.83	5.51
	4QNQ	6QGH	2.37	2.37	2.80	3.49
	Average		3.24	3.45	3.31	4.50
	Docking Accuracy		25	25	37.5	12.5
VINA	6QGH	4QNQ	1.53	1.53	1.71	3.83
	4QNQ	6QGH	2.31	1.67	4.69	3.22
	Average		1.92	1.60	3.20	3.52
	Docking Accuracy		75	100	50	25
VINARDO	6QGH	4QNQ	1.41	1.88	1.56	3.95
	4QNQ	6QGH	1.46	2.60	4.15	3.65
	Average		1.43	2.24	2.85	3.80
	Docking Accuracy		100	75	50	25

ECCD: experimental conformation cross-docking; ECCD: experimental conformation cross-docking after minimization; RCCD: random conformation cross-docking; docking; RCCD: random conformation cross-docking after minimization; VINA, VINARDO, AD4_SCORING are the scoring function available in smina; pdb entry code: PDB codes for Bcl-2 and Bcl-xL proteins co-crystallized with navitoclax

Table S14. *Docking energies (kcal/mol) for IS21, IS20, IS1, ISQ, ISP, IS9, IS27 and IS36 into wild type and three mutated Bcl-2 proteins. As comparison the SPR experimental KD$_1$ are also displayed.*

Cmpd	KD$_1$*	Docking energies (kcal/mol) into PDB IDs (BCL-2)			
		6O0K (WT)	6O0L (G101V)	6O0M (F104L)	6O0P(G101A)
IS21	0.19	-8.7	-8.9	-9.0	-8.7
IS20	0.32	-8.3	-7.2	-8.2	-8.3
IS1	0.48	-8.5	-7.5	-7.7	-8.4
ISQ	0.53	-8.5	-8.6	-8.6	-8.8
ISP	0.77	-8.3	-8.1	-8.6	-8.3
IS29	3.40	-8.1	-7.9	-8.2	-8.3
IS9	4.00	-7.4	-6.7	-7.0	-6.8
IS27	4.60	-7.9	-7.7	-8.4	-7.6

Table S15. *List of used combinations for the docking experiments.*

#	Docking Combination	Software	Scoring Function	Minimization feature
**1**	Smina/AD4_SCORING/Raw	Smina	Autodock 4	no
**2**	Smina/AD4_SCORING/Min	Smina	Autodock 4	yes
**3**	Smina/VINA/Raw	Smina	Vina	no
**4**	Smina/VINA/Raw	Smina	Vina	yes
**5**	Smina/VINARDO/Raw	Smina	Vinardo	no
**6**	Smina/VINARDO/Raw	Smina	Vinardo	yes
**7**	Plants/PLP	Plants	PLP	-
**8**	Plants/PLP95	Plants	PLP95	-
**9**	Plants/CHEMPLP	Plants	ChemPLP	-

Table S16. *PDB codes for the co-crystallized venetoclax/Bcl-2 complexes.*

PDB entry code	Mutation	Reference
6O0K	Wild Type	[12]
6O0L	G101V	[12]
6O0M	F104L	[12]
6O0P	G101A	[12]

Table S17. *Setting for Smina.*

```
autobox_ligand = reference.pdb
autobox_add = 5
cpu = 12
exhaustiveness = 32
min_rmsd_filter = 2
num_modes = 100
```

For the minimization feature the minimize_iters = 1000 key was used

Table S18. *Setting for Plants (example for chemplp scoring function).*

```
# scoring function and search settings
aco_ants 20
scoring_functionchemplp
outside_binding_site_penalty 50.0
enable_sulphur_acceptors 0
ligand_intra_score clash2
search_speed speed1
outside_binding_site_penalty 50.0
flip_amide_bonds 1
flip_planar_n 1
force_flipped_bonds_planarity 0
force_planar_bond_rotation 1
rescore_mode simplex
flip_ring_corners 0
chemplp_clash_include_14 1
chemplp_clash_include_HH 0
```

```
plp_steric_e -0.4
plp_burpolar_e -0.05
plp_hbond_e -2.0
plp_metal_e -4.0
plp_repulsive_weight 0.5
plp_tors_weight 1.0
chemplp_weak_cho 1
chemplp_charged_hb_weight 2.0
chemplp_charged_metal_weight 2.0
chemplp_hbond_weight -3.0
chemplp_hbond_cho_weight -3.0
chemplp_metal_weight -6.0
chemplp_plp_weight 1.0
chemplp_plp_steric_e -0.4
chemplp_plp_burpolar_e -0.1
chemplp_plp_hbond_e -0.1
chemplp_plp_metal_e -1.0
chemplp_plp_repulsive_weight 1.0
chemplp_tors_weight 2.0
chemplp_lipo_weight 0.0
chemplp_intercept_weight -20.0
# binding site definition
bindingsite_center 9.0 3.0 -7.0
bindingsite_radius 21.0
# output
write_protein_conformations 0
write_protein_bindingsite 0
write_protein_splitted 0
write_rescored_structures 1
write_multi_mol2 1
write_ranking_links 1
write_ranking_multi_mol2 0
write_per_atom_scores 1
write_merged_ligand 0
write_merged_protein 0
write_merged_water 0
keep_original_mol2_description 1
merge_multi_conf_output 1
merge_multi_conf_character .
# write single mol2 files (e.g. for RMSD calculation)
write_multi_mol2 0
write_ranking_links 1
# cluster algorithm
cluster_structures 100
cluster_rmsd 2.0
```

SUPPLEMENTARY FIGURES

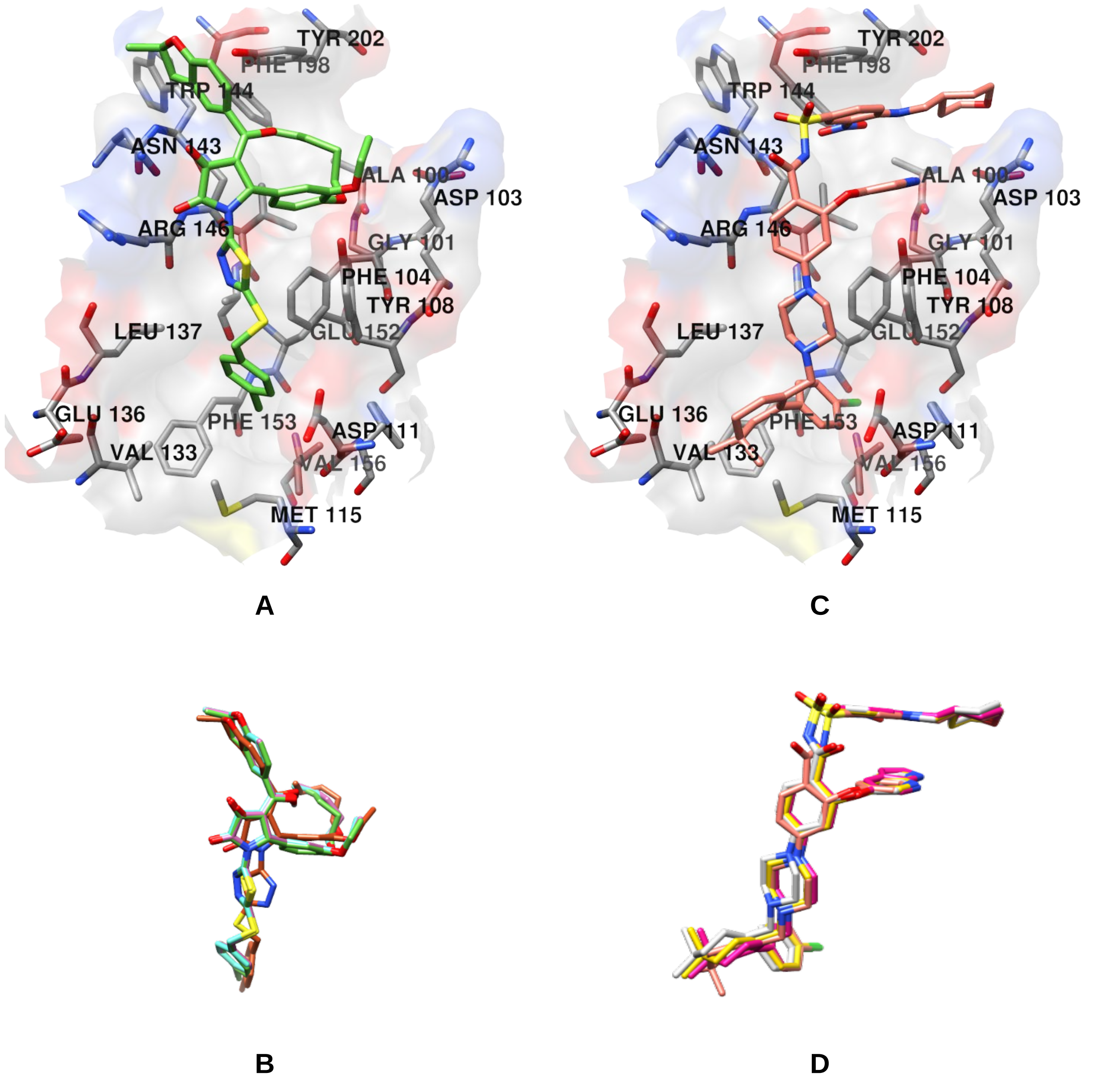

Figure S1. (**A**) Docked conformation of IS21 into the wild type Bcl-2. (**B**) structure-based superimposed IS21 docked conformation in the *wild* type and three mutated Bcl-2 proteins. (**C**) Venetoclax experimental bound conformation as in the wild type Bcl-2 (pdb id 6O0K). (**D**) structure-based overlapped experimental bound conformations of venetoclax as in *wild* type and three mutated Bcl-2 proteins (pdb ids: 6O0K (WT), 6O0L (G101V), 6O0M (F104L) and 6O0P(G101A).

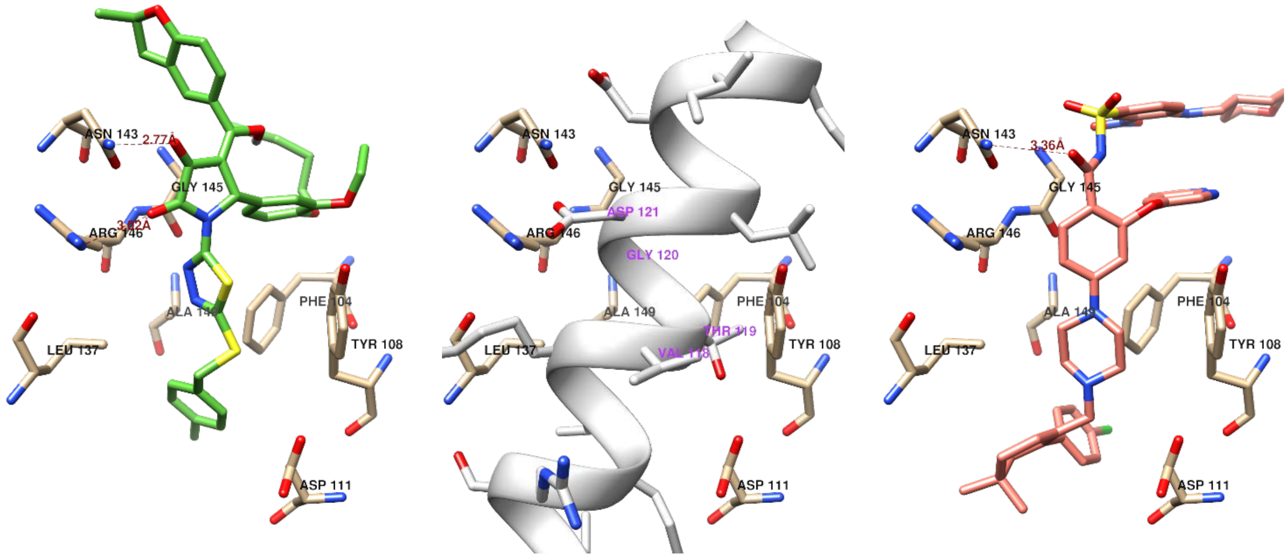

IS21 BH3 Venetoclax

Figure S2. Docked conformation of IS21 (green colored carbon atoms) into wild type Bcl-2 compared to experimental venetoclax bound conformation (orange colored carbon atoms) BH3 α-helix (light gray colored ribbon and carbon atoms) is also show as extracted from 5VAX pdb entry.

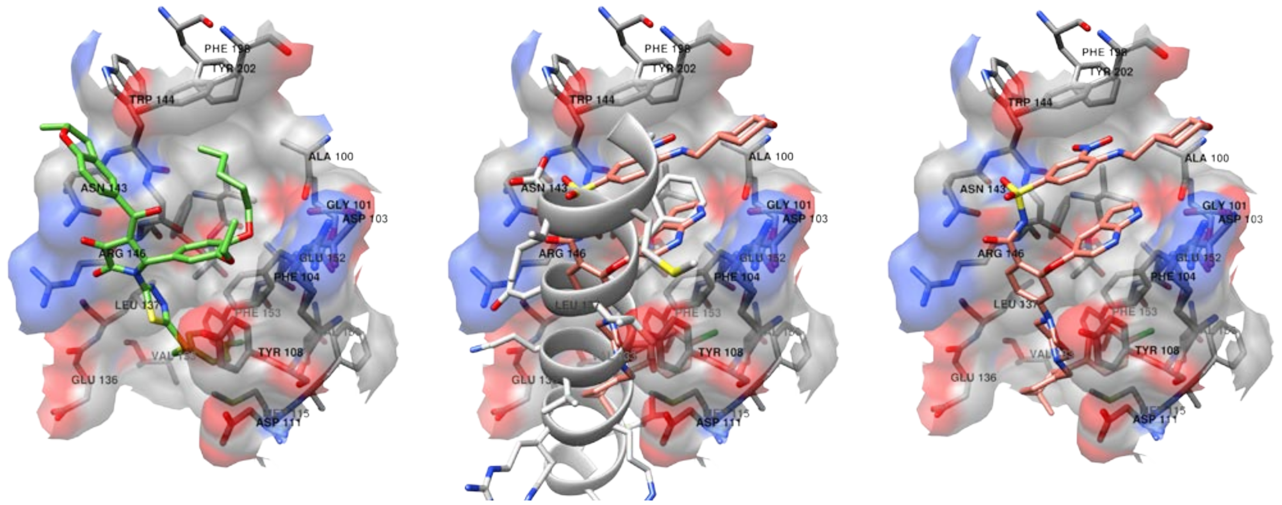

IS21 BH3 Venetoclax

Figure S3. Docked conformation of IS21 (green colored carbon atoms) into wild type Bcl-2 compared to experimental venetoclax bound conformation (orange colored carbon atoms and golden surface) as found in the 6O0k pdb entry. BH3 α-helix (light gray colored ribbon and carbon atoms) is also show as extracted from 5VAX pdb entry. The Bcl-2 is also showed as colored by atom type.

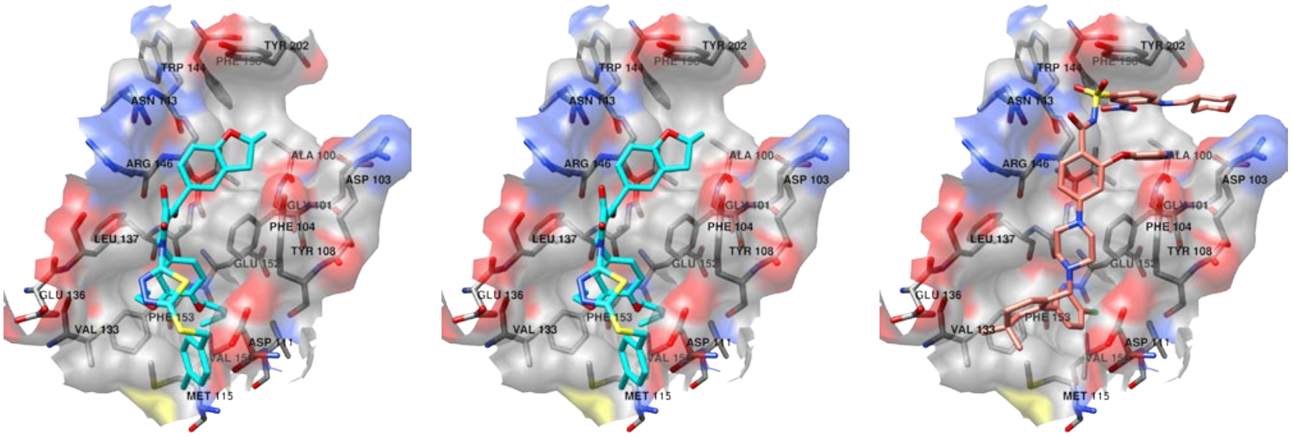

IS20 IS20 and venetoclax Venetoclax

Figure S4. Docked conformation of IS20 (cyan colored carbon atoms) into wild type Bcl-2 compared with the experimental bound conformation of venetoclax (orange colored carbon atoms) as found in the 6O0k pdb entry. The Bcl-2 is also showed as colored by atom type

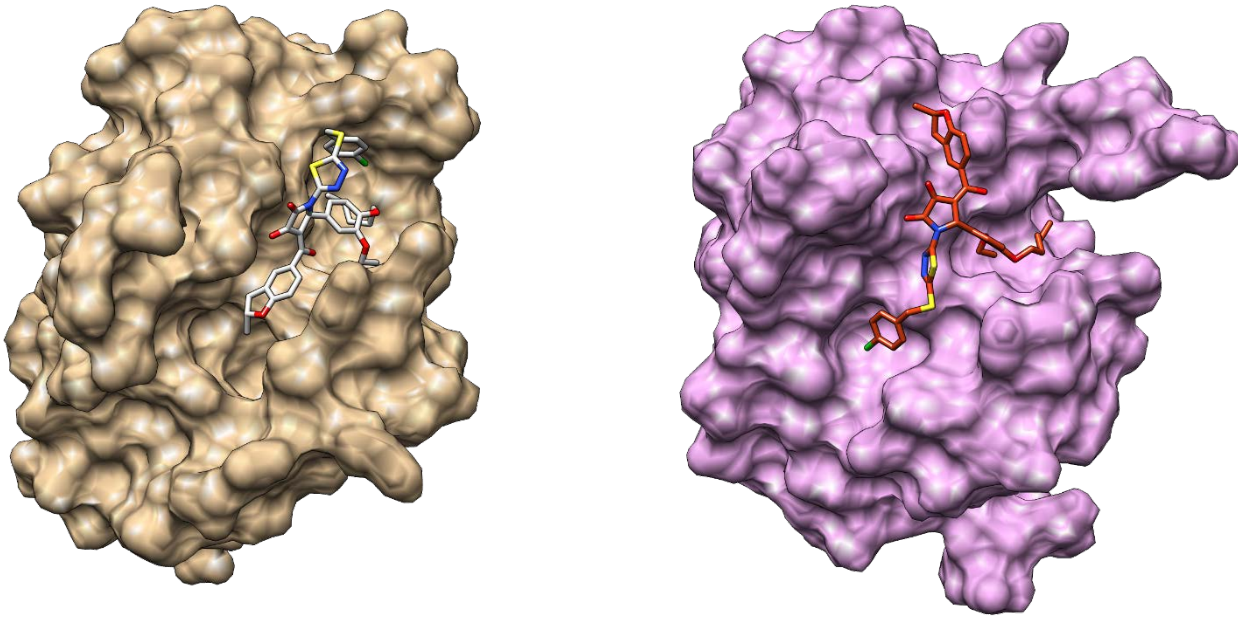

A **B**

Figure S5. (**A**) IS21 docked conformation into Bcl-2 protein extracted from 6QGH. (**B**) IS21 docked conformation into Bcl-2 protein extracted from 4BAS.

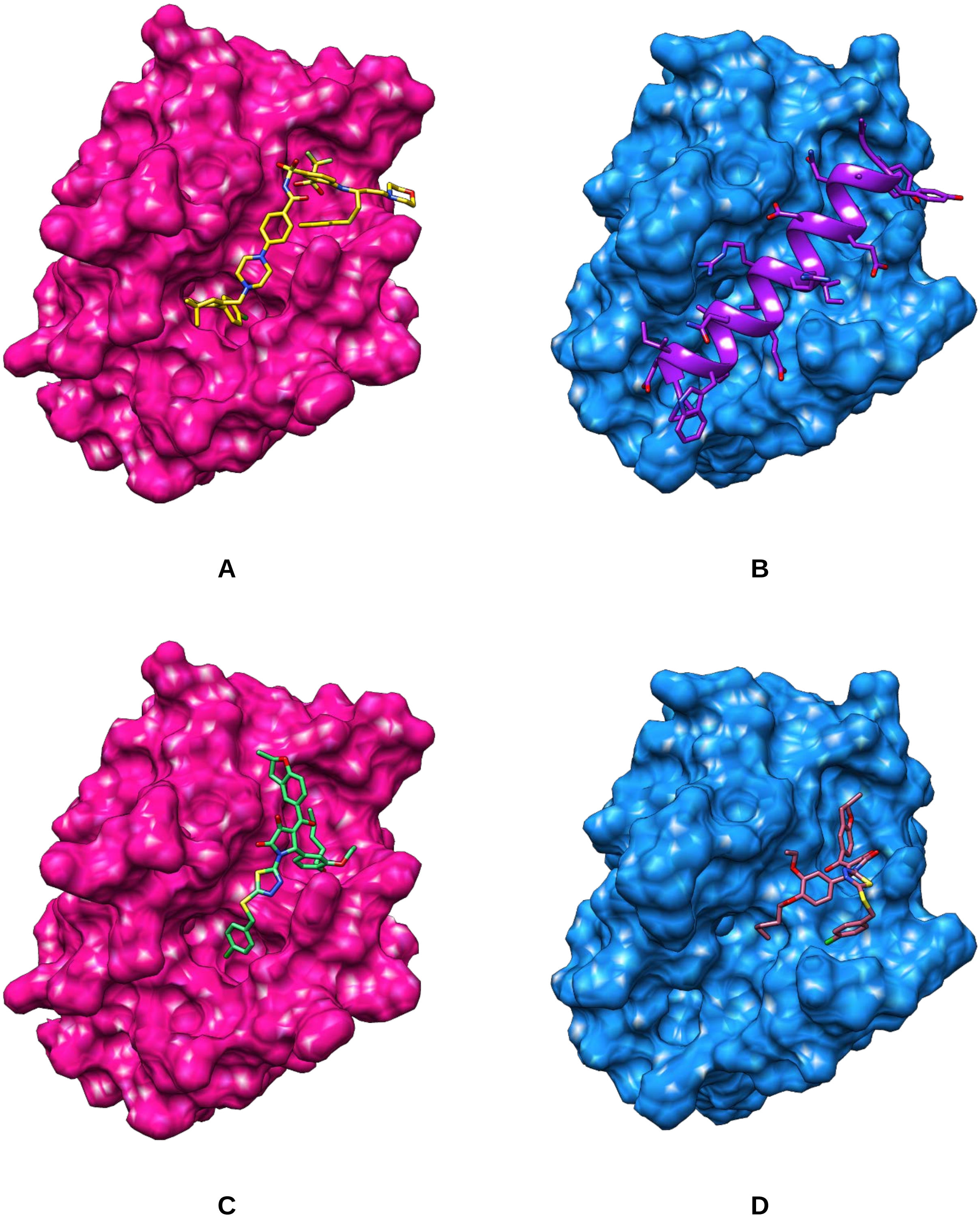

Figure S6. (**A**) Bcl-xL protein complexed with Navitoclax (pdb id 4QNQ). (**B**): Bcl-xL protein complexed with BIN BH3 (pdb id 4QVF). (**C**) IS21 docked conformation into Bcl-xL protein extracted from 4QNQ. (**D**) IS21 docked conformation into Bcl-xL protein extracted from 4QVF.

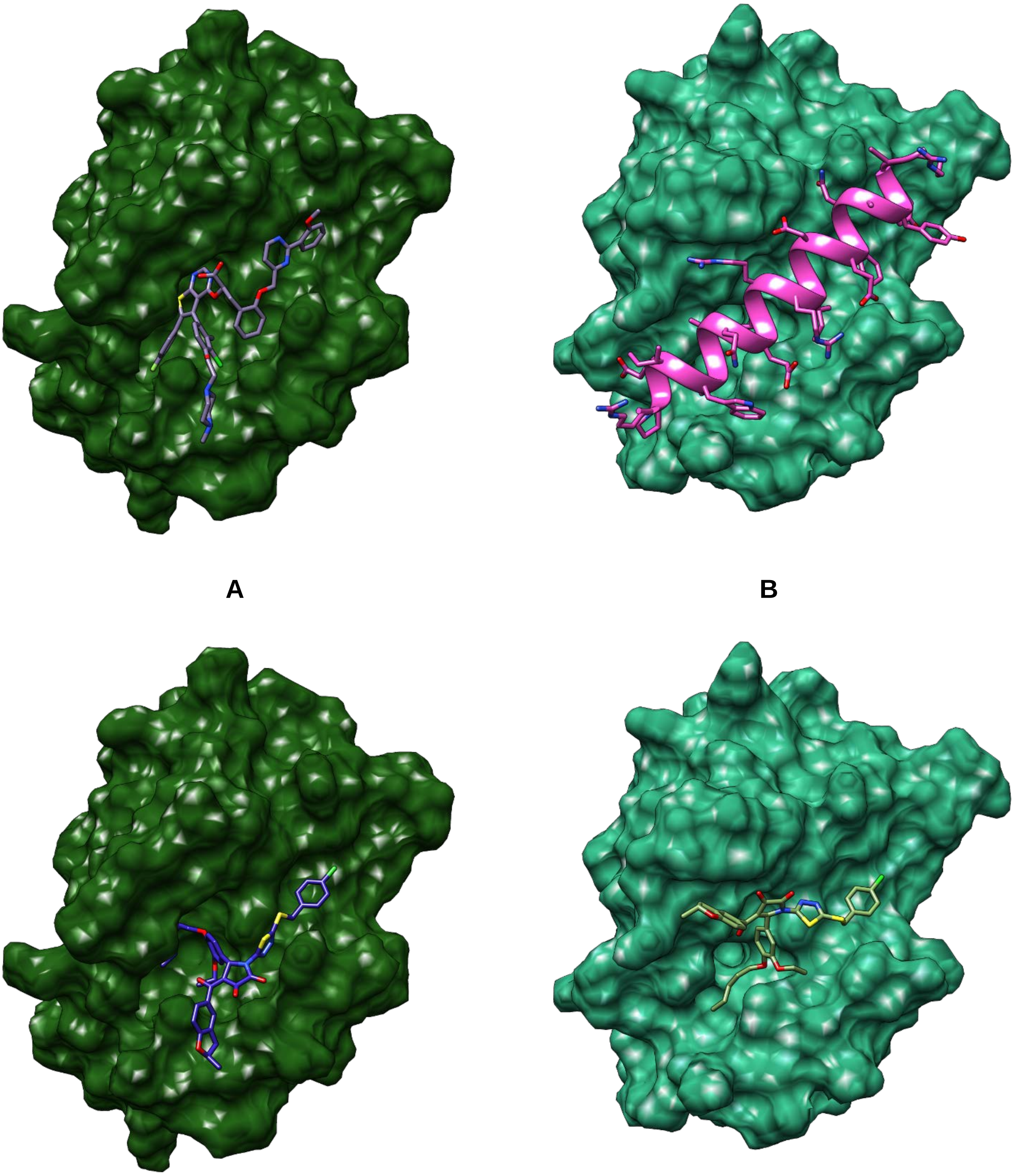

Figure S7. (**A**) Mcl-1 protein complexed with Navitoclax (pdb id 6YBL). (**B**) Mcl-1 protein complexed with BIN BH3 (pdb id 6QFI). (**C**) IS21 docked conformation into Bcl-2 protein extracted from 6YBL. (**D**) IS21 docked conformation into Mcl-1 protein extracted from 6QFI.

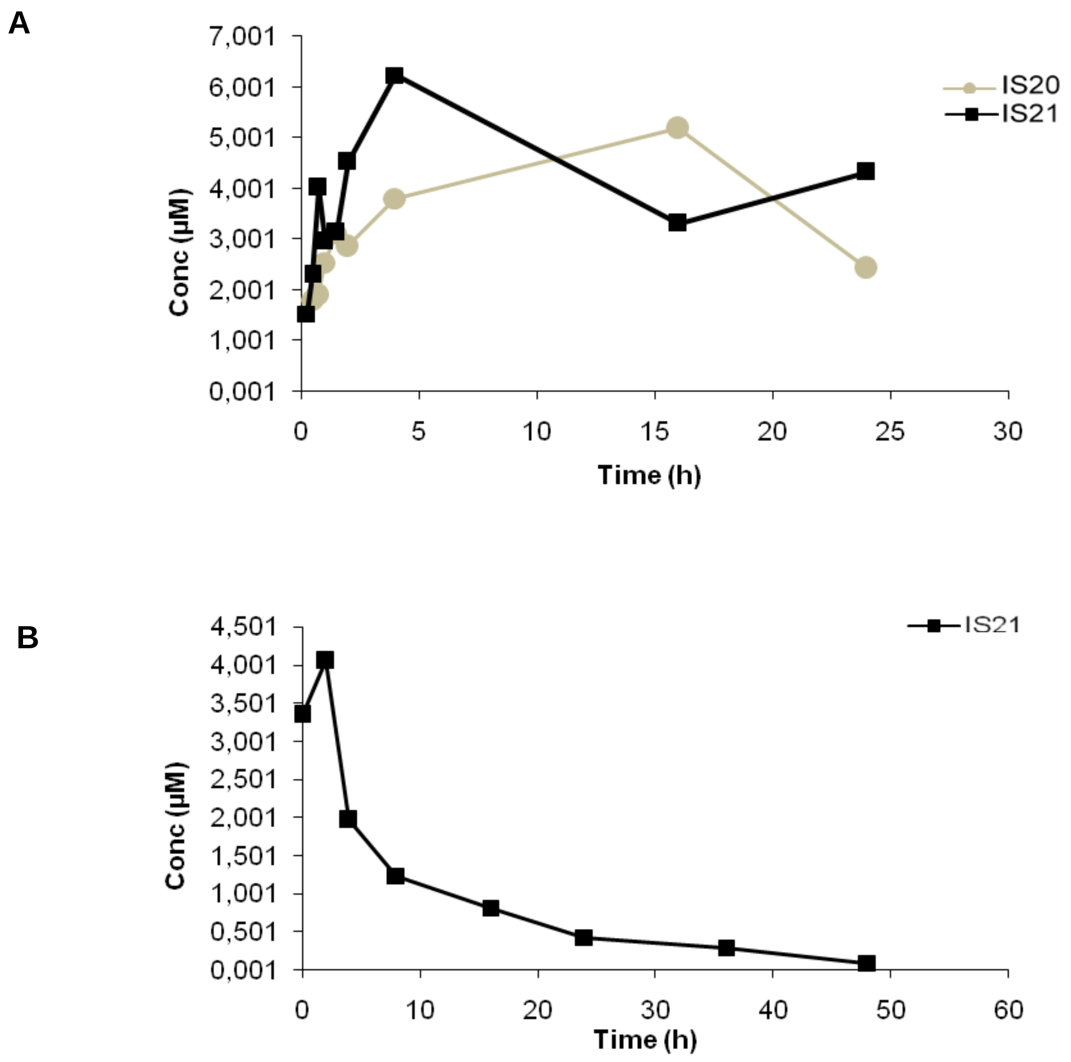

Figure S8. (**A**) Biopharmaceutical profile of IS20 (grey line) and IS21(black line). Graph showing the more rapid adsorption of IS21 respect to IS20 in mice plasma following drug administration at 100 mg/kg. (**B**) Biopharmaceutical profile of IS21. Graph showing the measure of IS21 in mice plasma following drug administration at 50 mg/kg. (**A-B**) Data are reported as mean ± SD peak of IS20 or IS21 (Conc. µM) at different times (n = 3).

A

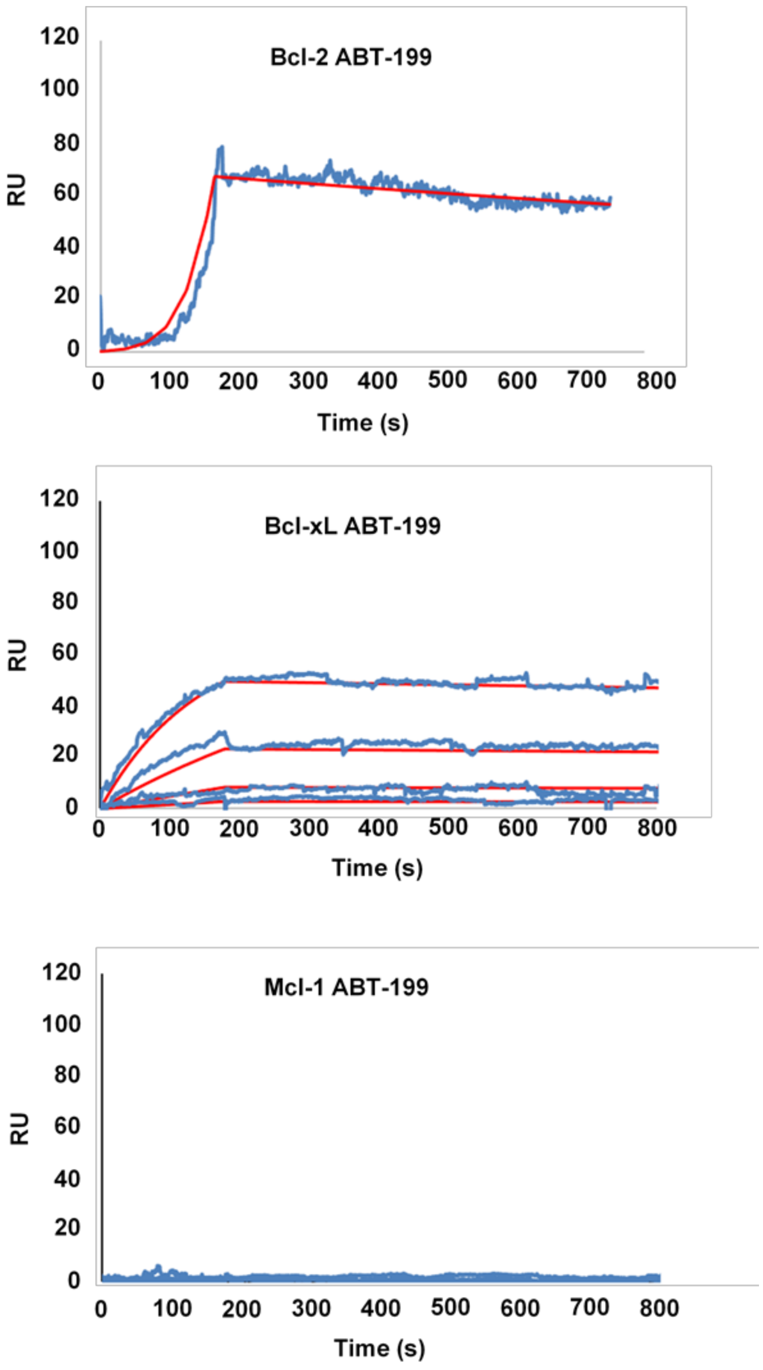
Bcl-2 ABT-199
Bcl-xL ABT-199
Mcl-1 ABT-199
RU
120
100
80
60
40
20
0
0 100 200 300 400 500 600 700 800
Time (s)

B

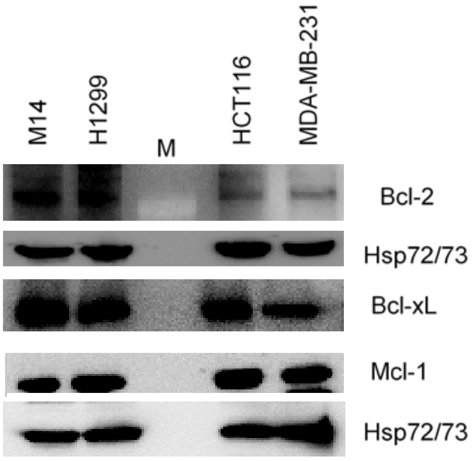
M14
H1299
M
HCT116
MDA-MB-231
Bcl-2
Hsp72/73
Bcl-xL
Mcl-1
Hsp72/73

Figure S9. (**A**) SPR experiments were carried out on Bcl-2, Bcl-xL and Mcl-1 (ligands) immobilized on COOH5 sensorchips, using venetoclax (ABT-199) as analyte. (**B**) Western blot analysis of Bcl-2, Bcl-xL and Mcl-1 proteins expression in M14, H1299, HCT116 and MDA-MB-231 cell lines. Reported images are representative of two independent experiments with similar results. HSP72/73 is shown as loading and transferring control.

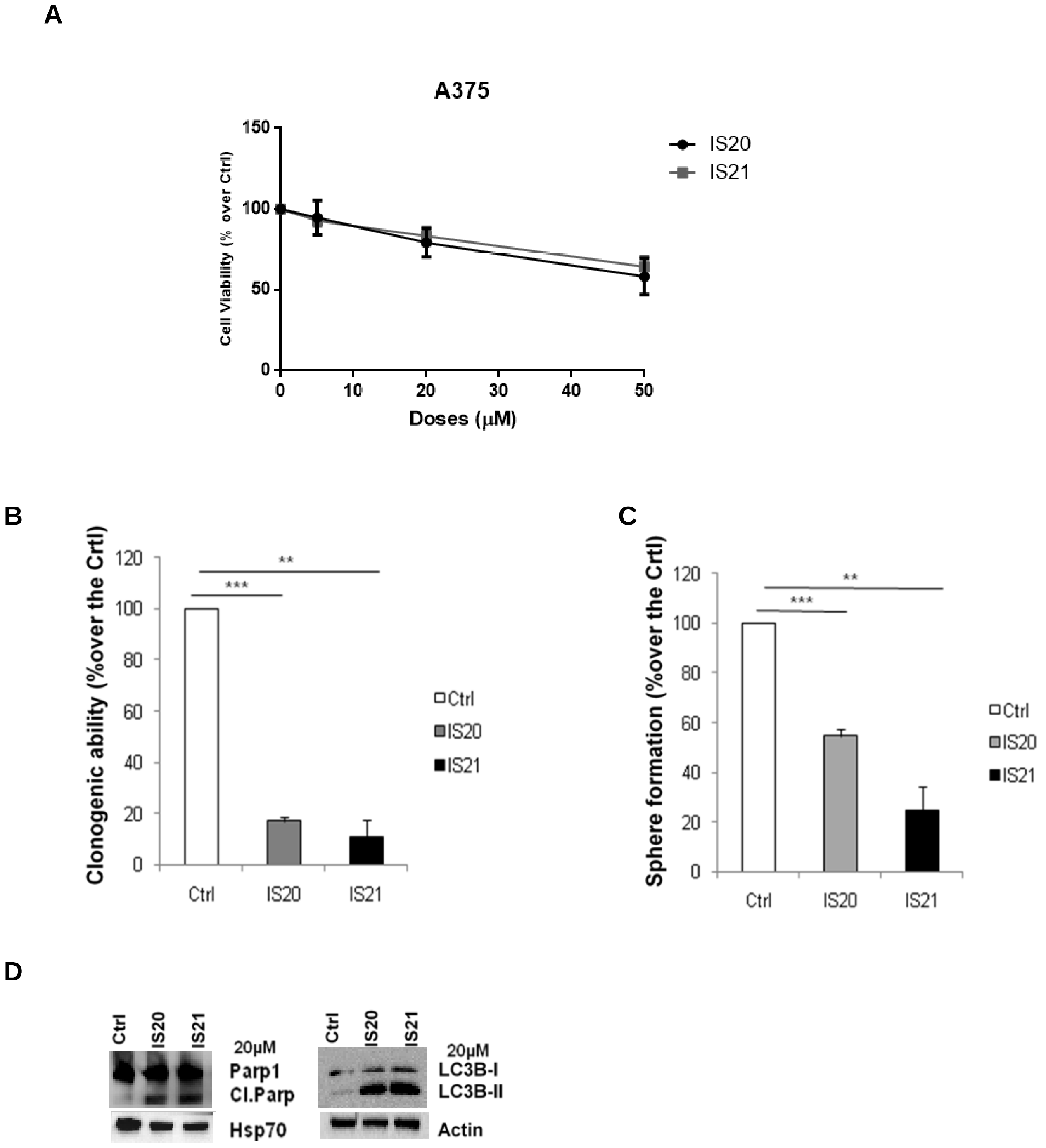

***Figure S10*. Effect of IS20 and IS21, on viability, clonogenic ability, sphere formation, apoptosis and autophagy of A375 cells (20 µM for 72 h).** (**A**) Analysis of cell viability by MTT assay after treatment with IS20 or IS21. The results are reported as "viability of treated cells/viability of control cells (Ctrl)" × 100. (**B**) Quantification of clonogenic ability after treatment with IS20 or IS21. Results are reported as percentage of clonogenicity of treated versus untreated cells (Ctrl). (**C**) Quantification of tumor sphere formation after treatment with IS20 or IS21. Results are reported as percentage of tumor sphere formation of treated versus untreated cells (Ctrl). (**B-C**) Data are reported as mean ± SD of three independent experiments. p-values were calculated between control (Ctrl) and treated cells, ** $p < 0.001$ and *** $p < 0.0001$. (**D**) Western blot analysis of PARP1 cleavage (cl. PARP) and LC3B-I and LC3B-II levels after treatment with IS20 or IS21. Reported images are representative of two independent experiments with similar results. HSP72/73 and Actin are shown as loading and transferring control.

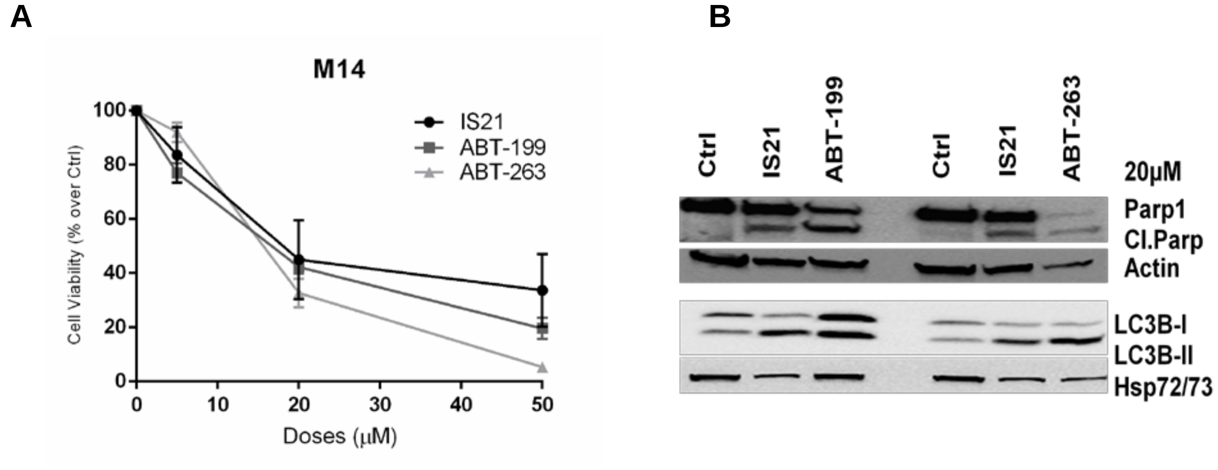

***Figure S11*. Effect of IS20, IS21, ABT-263 and ABT-199 on viability, apoptosis and autophagy of M14 cells (20 µM for 72 h).** (**A**) Analysis of cell viability by MTT assay in M14 cells treated with IS21, ABT-199 or ABT-263 (20 µM for 72 h). (**B**) Western blot analysis of PARP1 cleavage (cl. PARP) and LC3B-I and LC3B-II levels in M14 melanoma cell line treated with IS21, ABT-199 or ABT-263 (20 µM for 72 h). Reported images are representative of two independent experiments with similar results. HSP72/73 and Actin are shown as loading and transferring control. (**B-C**) The results are reported as "viability of treated cells/viability of control cells (Ctrl)" × 100. Data are reported as mean ± SD of three independent experiments.

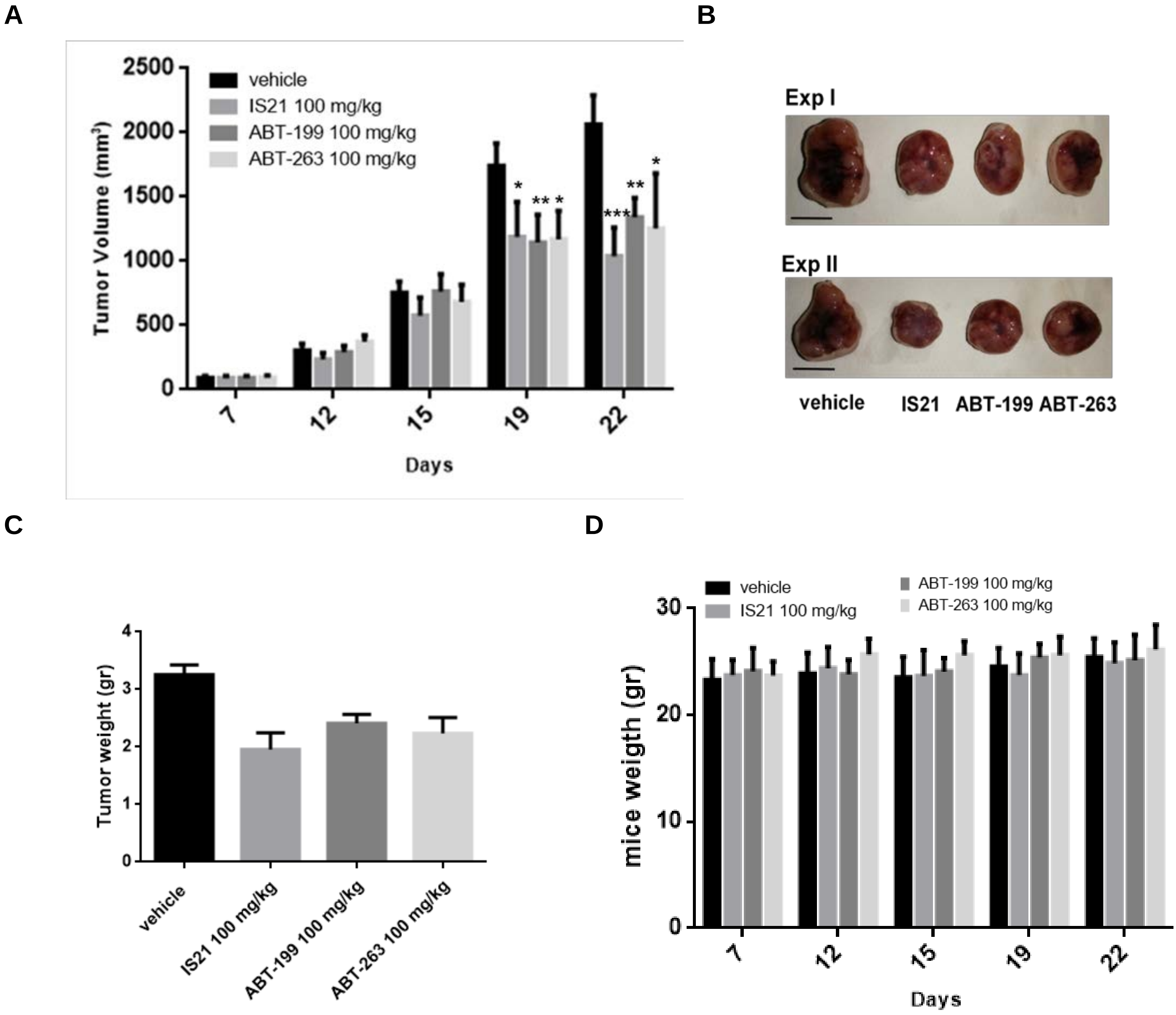

Figure S12*. *Effect of IS21, ABT-199 and ABT-263 on A375 in vivo tumor growth. (**A**) Analysis of *in vivo* tumor growth in nude mice injected with A375 cells and treated with vehicle, IS21, ABT-199 or with ABT-263 at the indicated concentrations for two weeks. Experiments were repeated twice. 4 animals/group. * $p < 0.01$, ** $p < 0.001$, *** $p < 0.0001$. (**B**) Representative images of explanted tumors of two independent experiments. The scale bar represents 1 cm. (**C**) Analysis of tumor weight after the *in vivo* experiment performed as reported in (A). ** $p \leq 0.001$ calculated as the mean of two experiments. (**D**) Analysis of mice weight after the *in vivo* experiment performed as reported in (A). (**A,C,D**) Data are reported as mean ± SD of two independent experiments.

References

1. Pedregosa F, Varoquaux G, Gramfort A, Michel V, Thirion B, Grisel O, et al. Scikit-learn: Machine Learning in Python. J Mach Learn Res 2011;12:2825-30.
2. Cao J, Kwong S, Wang R, Li X, Li K, Kong X. Class-Specific Soft Voting based Multiple Extreme Learning Machines Ensemble. Neurocomputing 2015;149:275-84.
3. Quiroga R, Villarreal MA. Vinardo: A Scoring Function Based on Autodock Vina Improves Scoring, Docking, and Virtual Screening. PLoS One 2016;11(5):e0155183.
4. Korb O, Stutzle T, Exner TE. Empirical scoring functions for advanced protein-ligand docking with PLANTS. J Chem Inf Model 2009;49(1):84-96.
5. Ragno R, Frasca S, Manetti F, Brizzi A, Massa S. HIV-reverse transcriptase inhibition: inclusion of ligand-induced fit by cross-docking studies. J Med Chem 2005;48(1):200-12.
6. Liu X, Zhang Y, Huang W, Tan W, Zhang A. Design, synthesis and pharmacological evaluation of new acyl sulfonamides as potent and selective Bcl-2 inhibitors. Bioorganic & Medicinal Chemistry 2018;26(2):443-54.
7. Nhu D, Lessene G, Huang DCS, Burns CJ. Small molecules targeting Mcl-1: the search for a silver bullet in cancer therapy. MedChemComm 2016;7(5):778-87.
8. Stazi G, Battistelli C, Piano V, Mazzone R, Marrocco B, Marchese S, et al. Development of alkyl glycerone phosphate synthase inhibitors: Structure-activity relationship and effects on ether lipids and epithelial-mesenchymal transition in cancer cells. Eur J Med Chem 2019;163:722-35.
9. Sabatino M, Rotili D, Patsilinakos A, Forgione M, Tomaselli D, Alby F, et al. Disruptor of telomeric silencing 1-like (DOT1L): disclosing a new class of non-nucleoside inhibitors by means of ligand-based and structure-based approaches. J Comput Aided Mol Des 2018;32(3):435-58.
10. Mladenovic M, Patsilinakos A, Pirolli A, Sabatino M, Ragno R. Understanding the Molecular Determinant of Reversible Human Monoamine Oxidase B Inhibitors Containing 2H-Chromen-2-One Core: Structure-Based and Ligand-Based Derived Three-Dimensional Quantitative Structure-Activity Relationships Predictive Models. J Chem Inf Model 2017;57(4):787-814.
11. Ragno R, Ballante F, Pirolli A, Wickersham RB, 3rd, Patsilinakos A, Hesse S, et al. Vascular endothelial growth factor receptor-2 (VEGFR-2) inhibitors: development and validation of predictive 3-D QSAR models through extensive ligand- and structure-based approaches. J Comput Aided Mol Des 2015;29(8):757-76.
12. Birkinshaw RW, Gong JN, Luo CS, Lio D, White CA, Anderson MA, et al. Structures of BCL-2 in complex with venetoclax reveal the molecular basis of resistance mutations. Nat Commun 2019;10(1):2385.
13. Daina A, Michielin O, Zoete V. SwissADME: a free web tool to evaluate pharmacokinetics, drug-likeness and medicinal chemistry friendliness of small molecules. Sci Rep 2017;7:42717.
